# Supplementary material for: Antisense Therapy for a Common Corneal Dystrophy Ameliorates TCF4 Repeat Expansion-Mediated Toxicity
Source: Am J Hum Genet. 2018 Mar 8;102(4):528–39. doi: 10.1016/j.ajhg.2018.02.010 (PMC5985359; doi:10.1016/j.ajhg.2018.02.010)
Supplement: Document S1. Figures S1–S6 and Tables S1–S9 [file mmc1.pdf]

The American Journal of Human Genetics, Volume *102*

Supplemental Data

Antisense Therapy for a Common Corneal Dystrophy Ameliorates *TCF4* Repeat Expansion-Mediated Toxicity

Christina Zarouchlioti, Beatriz Sanchez-Pintado, Nathaniel J. Hafford Tear, Pontus Klein, Petra Liskova, Kalyan Dulla, Ma'ayan Semo, Anthony A. Vugler, Kirithika Muthusamy, Lubica Dudakova, Hannah J. Levis, Pavlina Skalicka, Pirro Hysi, Michael E. Cheetham, Stephen J. Tuft, Peter Adamson, Alison J. Hardcastle, and Alice E. Davidson

Supplemental Data

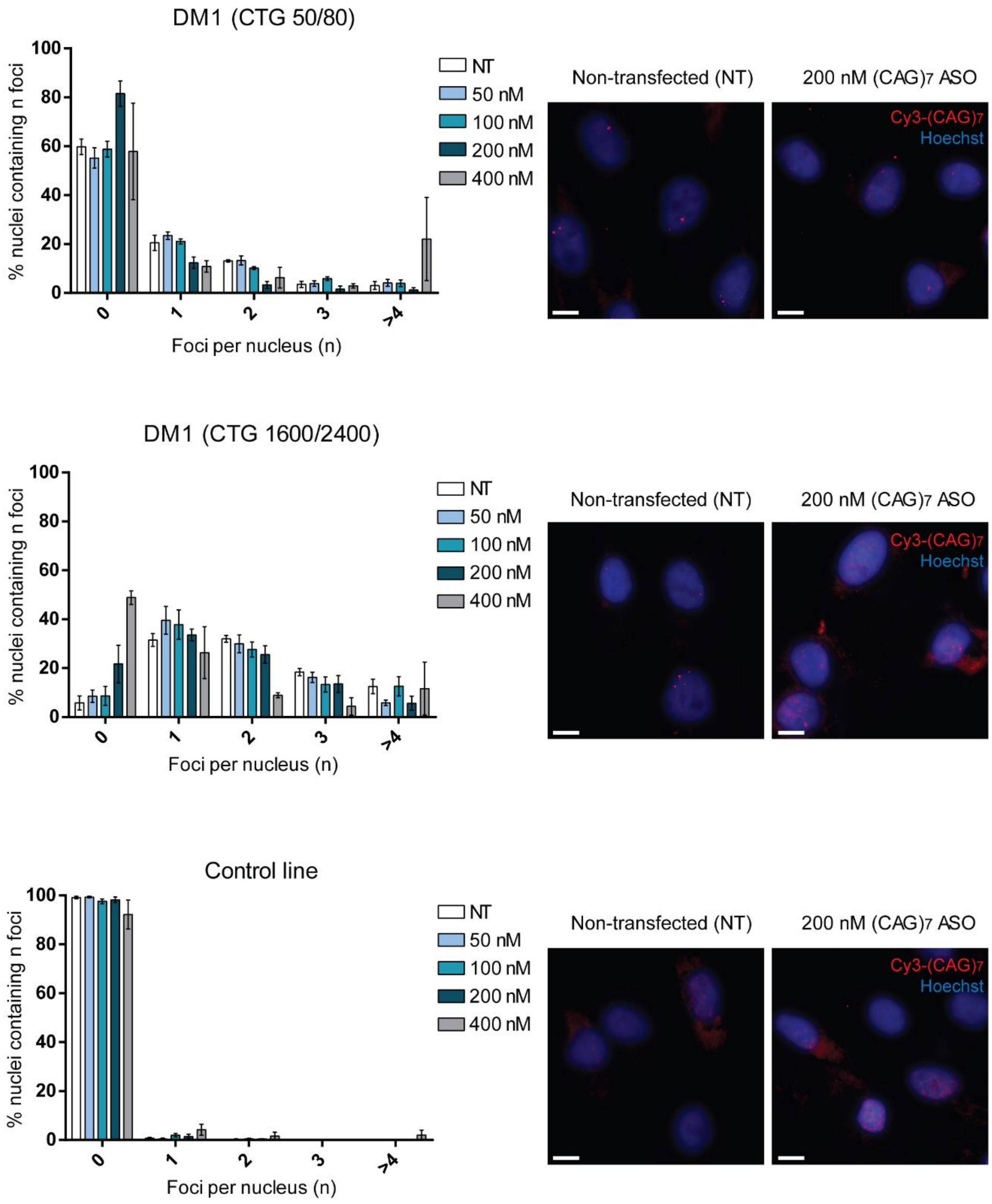

Figure S1. Optimization of antisense oligo (ASO) concentration used for *in vitro* transfections. Fibroblasts isolated from myotonic dystrophy type 1 (DM1) subjects harboring 50/80 CTG repeats, 1600/2400 CTG repeats, and control fibroblasts noted GM03991, GM04602, and GM04603 respectively (Coriell Institute) were transfected with 2'Ome-PS-$(CAG)_7$ ASO at indicated concentrations using Turbofect (ThermoFisher Scientific). Twenty-four hours post transfection fluorescence *in situ* hybridization (FISH) was performed and RNA foci were quantified using CellProfiler (Broad Institute). A concentration of 200 nM 2'Ome-PS-$(CAG)_7$ ASO was selected to be optimal, on the basis for these data, for future ASO transfection experiments in corneal endothelial cells (CECs). Scale bars, 10 µm.

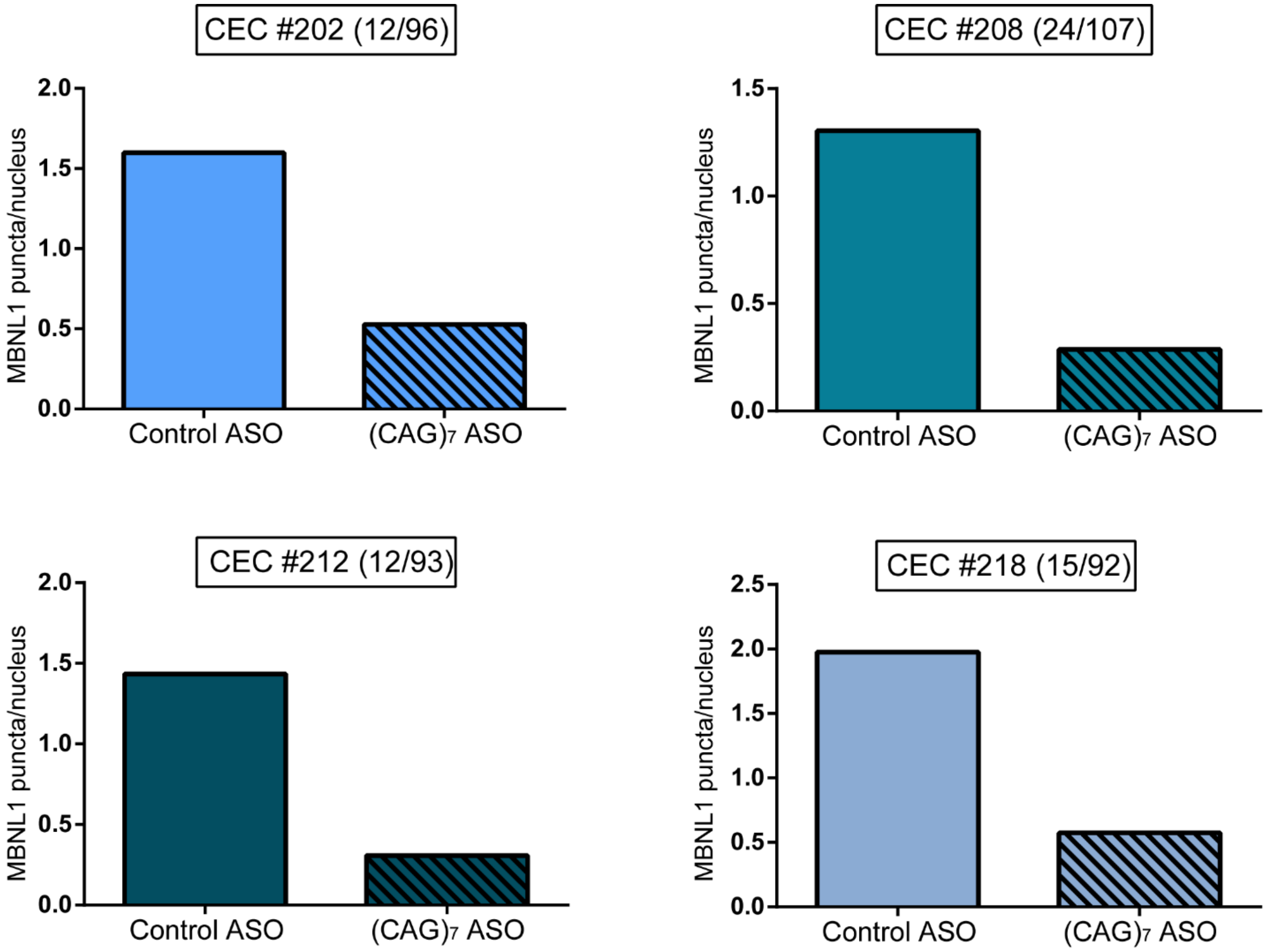

Figure S2. Quantification of nuclear MBNL1-positive puncta post antisense oligo (ASO) treatments. The result of 4 independent Fuchs endothelial corneal dystrophy (FECD) case-derived CEC lines, where the numbers of MBNL1 puncta were quantified after treatment with the control and $(CAG)_7$ ASOs. CTG18.1 genotypes are shown in parentheses for each line investigated.

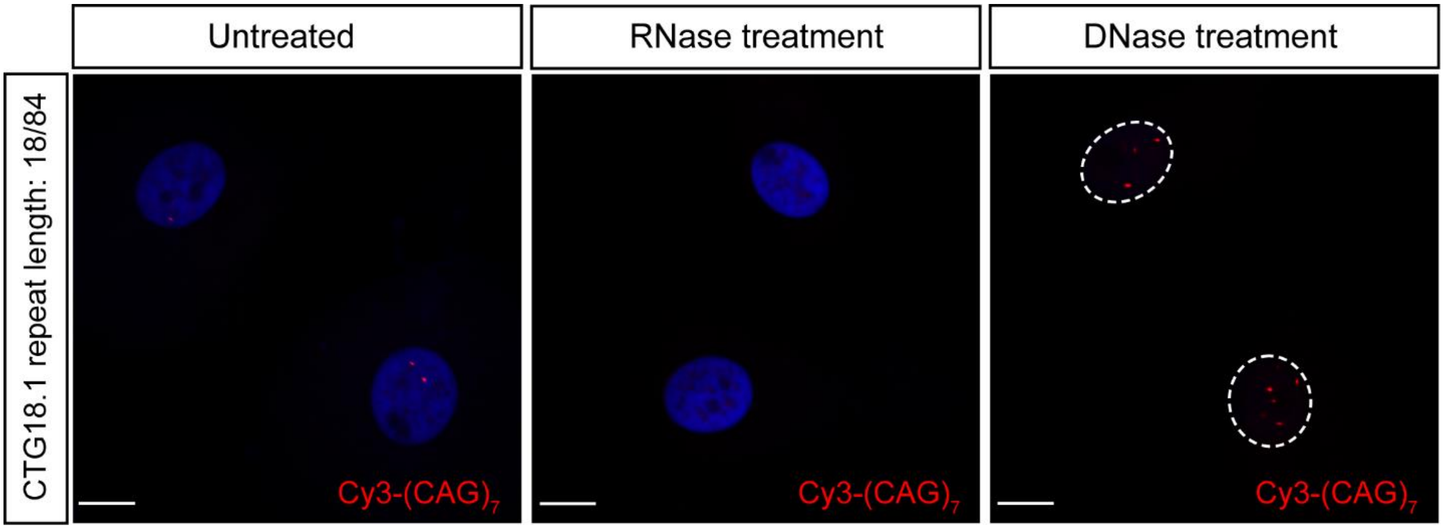

Figure S3. Fluorescence *in situ* hybridization (FISH) protocol detects RNA-specific CUG foci. Sense RNA foci detection with Cy3-$(CAG)_7$ probe in untreated, RNase treated and DNase treated FECD case-derived CECs with an expanded *TCF4* CTG18.1 allele (18/84). Scale bars, 10 µm.

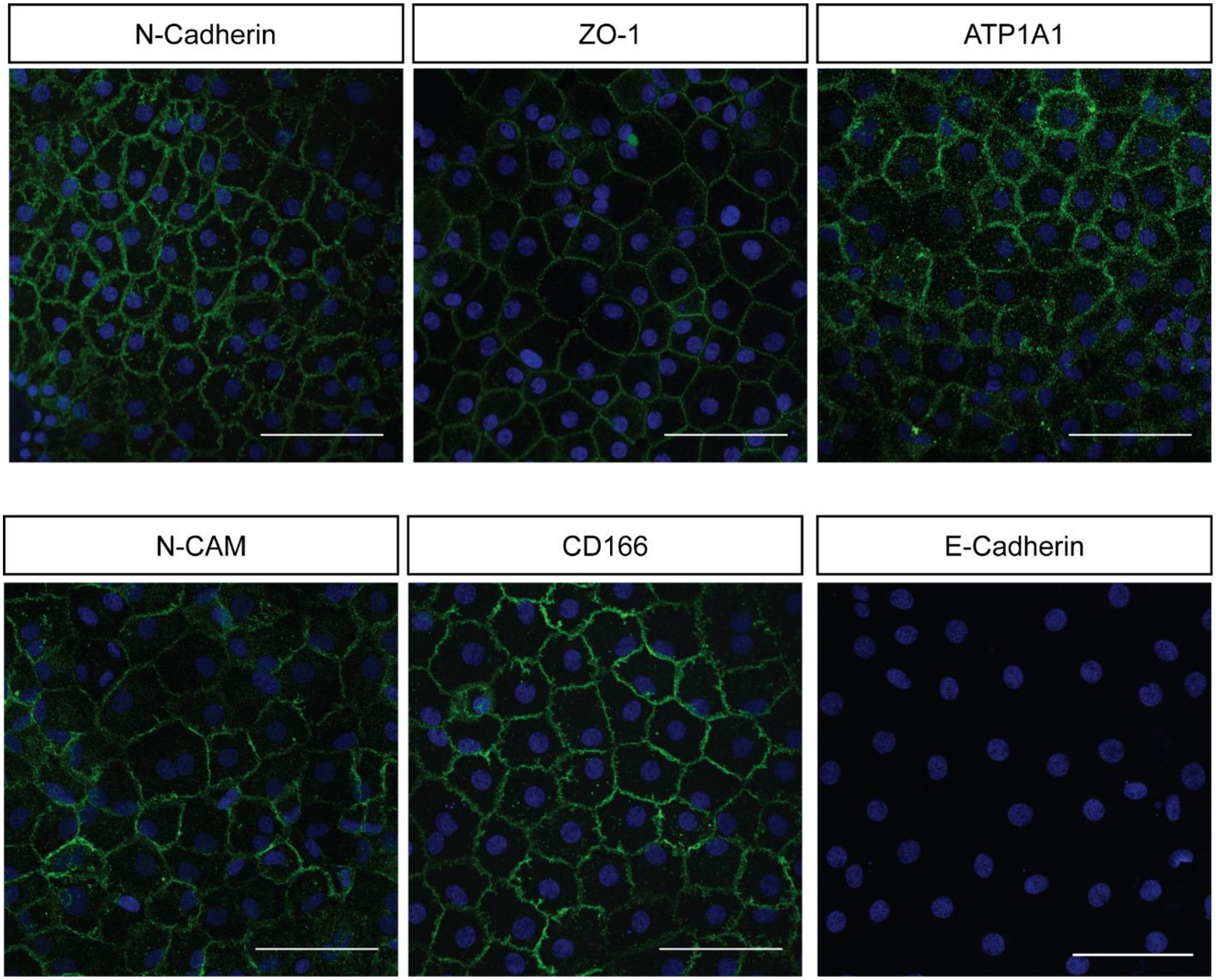

Figure S4. Primary corneal endothelial cell (CEC) cultures display distinctive corneal endothelial polygonal morphology and display markers indicative of endothelial cell status. Endothelial markers N-Cadherin, ZO-1, ATP1A1, N-CAM and CD166 were observed and the epithelial marker E-Cadherin was absent in CEC lines derived from healthy tissue. Scale bars, 100 µm.

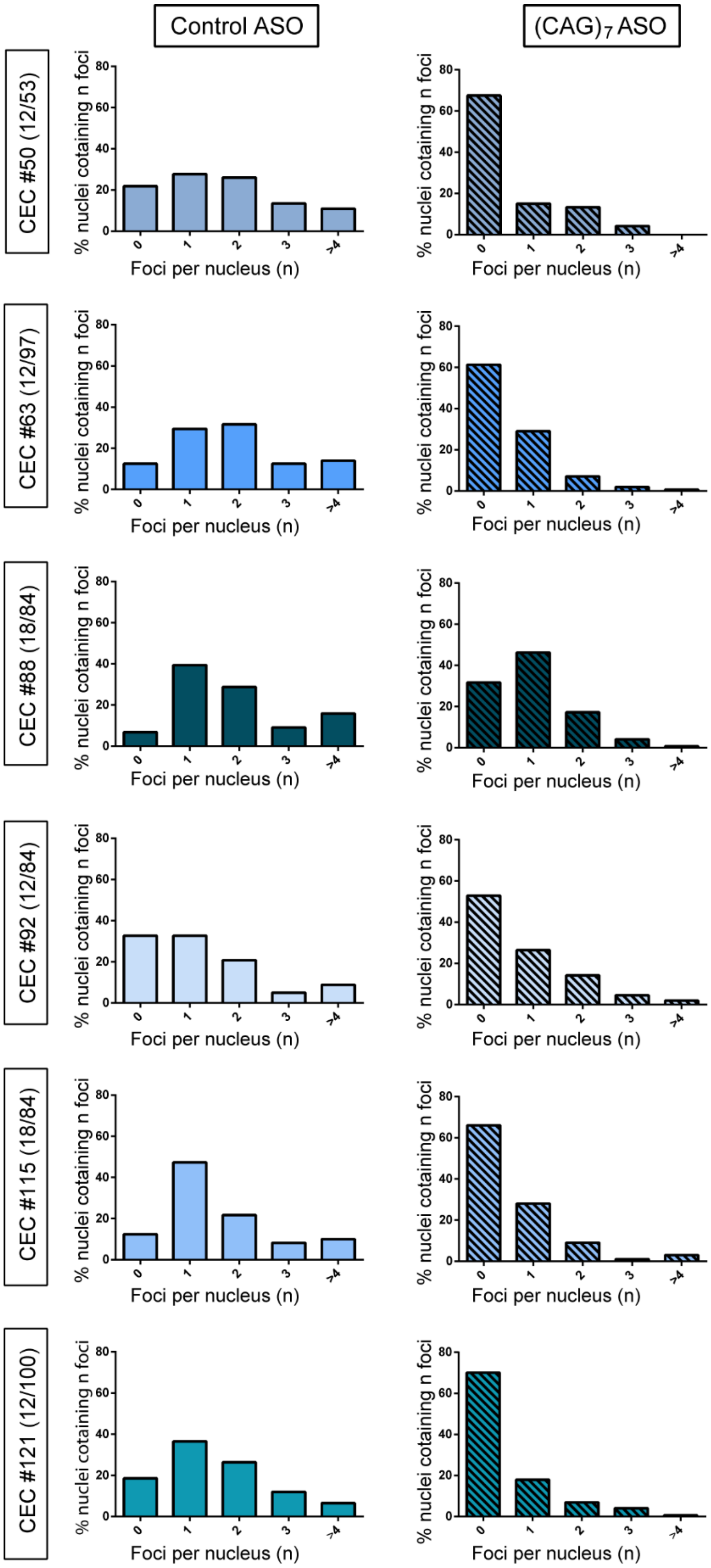

Figure S5. Antisense oligo (ASO)-mediated treatment of 6 independent FECD case-derived corneal endothelial cell (CEC) lines. The effect of the $(CAG)_7$ ASO treatment on foci incidence in each of the 6 independent FECD case-derived cell lines investigated is shown. CTG18.1 genotypes are shown in parentheses for each line investigated.

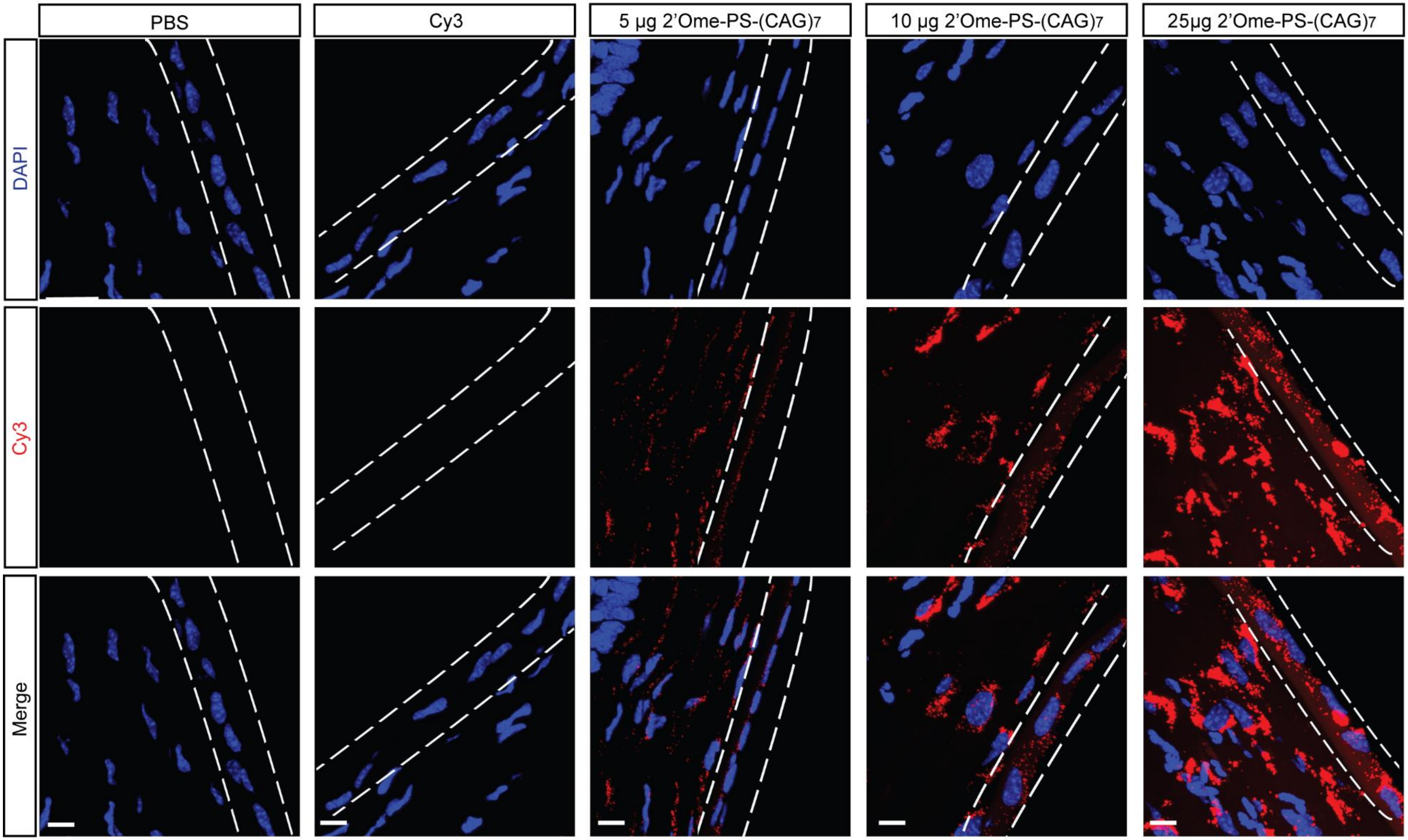

Figure S6. Visualization of Cy3-labelled 2'Ome-PS-$(CAG)_7$ antisense oligonucleotide (ASO) in corneal tissue following intraocular administration. C57Bl6 mice were intravitreally injected with 0.025, 0.01 or 0.005 mg of Cy-3 labelled 2'Ome-PS-$(CAG)_7$ ASO, free Cy3 dye alone (identical molar amount to the ASO), or PBS. Animals were sacrificed 48h post injection. Eyes were enucleated and immersion fixed in paraformaldehyde. Slides were prepared and immediately viewed on a confocal fluorescent microscope after counterstaining for cell nuclei detection with DAPI (blue). The location of the corneal endothelium is denoted by dashed lines. Scale bars, 20 µm.

Table S1. Summary of antibodies used in this study.

Target protein	Source	Dilution	Reference
ZO-1	Mouse	1:500	Invitrogen
ATP1A1	Mouse	1:50	Developmental studies Hybridoma Bank (a6F)
N-Cadherin	Rabbit	1:100	Abcam (ab12221)
N-CAM	Rabbit	1:100	Cell signaling 3606s
CD166	Mouse	1:200	Pharmingen (BD) 559260
E-Cadherin	Rat	1:500	Abcam (ab11512)
MBNL1	Rabbit	1:2000	Lin et al, 2006 [27]
MBNL2	Mouse	1:100	Santa Cruz (sc-365104)

Table S2. Primers used to detect alternative splice events by reverse transcriptase (RT)-PCR.

Primer	Sequence 5'-3'	Amplicon sizes (bp)	Optimized annealing temperature (^{0}C)
*MBNL1*_F	TGACACCAATGACAACACAGTC	383/329	65
*MBNL1*_ R	CTGGTGGGAGAAATGCTGTA		
*MBNL2*_ F	ACAGCACCATGATCGACACAAG	276/222	64
MBNL2 _R	AAGGATGAAGAGCACCAGGG		
*NUMA1*_F	TACGTGCTGATGCTGAGACC	305/263	64
*NUMA1*_R	CTTGCTGGCTTGGTCAGAGT		

Table S3: Fibroblast, and case matched corneal endothelial cell (CEC), lines investigated by fluorescence *in situ* hybridization (FISH). NA, not available. NI, not investigated. CTG18.1 repeat lengths were confirmed to be concordant between blood and fibroblast-derived gDNA samples.

Fibroblast line	Case matched CEC line	CTG18.1 genotype	CUG RNA foci detected in fibroblasts?	CUG RNA foci detected in CECs?
#F1	NA	31/69	No	NI
#F2	NA	12/53	No	NI
#F3	NA	84/108	No	NI
#F4	#43	23/68	No	Yes
#F5	#47	23/70	No	Yes
#F6	#31	12/77	No	Yes

Table S4: CTG18.1 genotype of FECD case-derived corneal endothelial cell (CEC) lines investigated by fluorescence *in situ* hybridization (FISH). Cell lines are ordered based on CTG18.1 genotype status in ascending order. Genotypes were generated using case matched whole blood-derived genomic DNA samples.

CEC line	CTG18.1 genotype	CUG RNA foci detected?
#94	12/12	No
#139	12/12	No
#42	12/15	No
#86	12/15	No
#32	12/16	No
#46	26/27	No
#190	15/16	No
#211	12/23	No
#61	18/31	No
#181	25/31	Yes
#50	12/53	Yes
#136	12/58	Yes
#8	16/62	Yes
#38	16/62	Yes
#152	15/68	Yes
#43	23/68	Yes
#33	16/70	Yes
#47	23/70	Yes
#120	23/70	Yes
#70	12/74	Yes
#49	12/76	Yes
#192	25/76	Yes
#31	12/77	Yes
#193	25/79	Yes
#25	24/80	Yes
#29	16/81	Yes
#37	12/82	Yes
#92	12/84	Yes
#88	18/84	Yes
#115	18/84	Yes
#34	12/87	Yes
#137	16/88	Yes
#63	12/97	Yes
#67	12/98	Yes
#121	12/100	Yes
#44	12/126	Yes

Table S5: FECD case-derived corneal endothelial cell (CEC) lines used for dual fluorescence *in situ* hybridization (FISH) and immunocytochemistry (ICC) experiments. NI, not investigated, E, expanded, NE, non-expanded.

Case derived CEC line	CTG18.1 genotype	CTG18.1 Genotype Classification	CUG RNA foci detected?	MBNL1 sequestered?	MBNL2 sequestered?
#121	12/100	E	Yes	Yes	NI
#190	15/16	NE	No	No	NI
#192	25/76	E	Yes	Yes	NI
#193	25/79	E	Yes	Yes	NI
#204	12/99	E	Yes	NI	Yes
#206	25/76	E	Yes	NI	Yes
#211	12/23	NE	No	NI	No
#226	12/18	NE	No	No	NI

Table S6: FECD case-derived corneal endothelial cell (CEC) lines used for transcriptomic analysis. E, expanded, NE, non-expanded.

Case matched CEC line	CTG18.1 genotype	CTG18.1 Genotype Classification
#138	16/84	E
#140	12/106	E
#149	16/93	E
#154	12/81	E
#125	13/18	NE
#141	18/24	NE
#145	22/28	NE

Table S7: FECD case-derived corneal endothelial cell (CEC) lines used for antisense oligonucleotide (ASO) treatment experiments. E, expanded.

Case matched CEC line	CTG18.1 genotype	CTG18.1 Genotype Classification
#88	18/84	E
#92	12/84	E
#115	18/84	E
#121	12/100	E
#50	12/53	E
#63	12/97	E

Table S8: FECD case-derived corneal endothelial cell (CEC) lines used to assess MBNL1 localization, following antisense oligonucleotide (ASO) treatment. E, expanded.

Case matched CEC line	CTG18.1 genotype	CTG18.1 Genotype Classification
#202	12/96	E
#208	24/107	E
#212	12/93	E
#218	15/92	E

Table S9: FECD case-derived corneal endothelial cell (CEC) lines used for transcriptomic analysis, following antisense oligonucleotide (ASO) treatment. E, expanded.

Case matched CEC line	CTG18.1 genotype	CTG18.1 Genotype Classification
#162	71/113	E
#173	16/72	E
#179	15/61	E
#210	14/80	E
#215	12/73	E
#216	18/91	E
#219	18/88	E
#220	15/103	E
#223	12/63	E
#224	12/89	E
