# Supplementary material for: Antisense Therapy for a Common Corneal Dystrophy Ameliorates TCF4 Repeat Expansion-Mediated Toxicity
Source: Am J Hum Genet. 2018 Mar 8;102(4):528–39. doi: 10.1016/j.ajhg.2018.02.010 (PMC5985359; doi:10.1016/j.ajhg.2018.02.010)
Supplement: Document S2. Article plus Supplemental Data [file mmc2.pdf]

ARTICLE

Antisense Therapy for a Common Corneal Dystrophy Ameliorates *TCF4* Repeat Expansion-Mediated Toxicity

Christina Zarouchlioti,[1,8] Beatriz Sanchez-Pintado,[1,8] Nathaniel J. Hafford Tear,[1,8] Pontus Klein,[2] Petra Liskova,[3,4] Kalyan Dulla,[2] Ma'ayan Semo,[1] Anthony A. Vugler,[1] Kirithika Muthusamy,[1,5] Lubica Dudakova,[3] Hannah J. Levis,[6] Pavlina Skalicka,[3,4] Pirro Hysi,[7] Michael E. Cheetham,[1] Stephen J. Tuft,[1,5] Peter Adamson,[2,9] Alison J. Hardcastle,[1,9] and Alice E. Davidson[1,9,*]

Fuchs endothelial corneal dystrophy (FECD) is a common disease for which corneal transplantation is the only treatment option in advanced stages, and alternative treatment strategies are urgently required. Expansion ($\geq$50 copies) of a non-coding trinucleotide repeat in *TCF4* confers >76-fold risk for FECD in our large cohort of affected individuals. An FECD subject-derived corneal endothelial cell (CEC) model was developed to probe disease mechanism and investigate therapeutic approaches. The CEC model demonstrated that the repeat expansion leads to nuclear RNA foci, with the sequestration of splicing factor proteins (MBNL1 and MBNL2) to the foci and altered mRNA processing. Antisense oligonucleotide (ASO) treatment led to a significant reduction in the incidence of nuclear foci, MBNL1 recruitment to the foci, and downstream aberrant splicing events, suggesting functional rescue. This proof-of-concept study highlights the potential of a targeted ASO therapy to treat the accessible and tractable corneal tissue affected by this repeat expansion-mediated disease.

Introduction

Fuchs endothelial corneal dystrophy (FECD [MIM: 613267]) is a common, degenerative, age-related condition that usually presents during the fifth to sixth decade. The disease primarily affects the posterior cornea with the hallmark being the formation of focal excrescences of Descemet membrane (DM) termed "guttae." Isolated guttae are common in the elderly and a case definition for FECD usually requires that the guttae are confluent. When assessed with specular microscopy, the prevalence of guttae is higher in white (11.2% >55 years) than East Asian (5.5% >50 years) populations and higher in females (5.5%–11%) than males (1.5%–7%).[1,2] An estimate of the prevalence of confluent guttae in individuals of white or black ethnicity >50 years with assessment by slit-lamp biomicroscopy reported a figure of 4.5%.[3] Confluent guttae can be associated with a loss of corneal endothelial cell density to a critical stage when the remaining endothelium is unable to maintain appropriate stromal dehydration leading to fluid accumulation, painful epithelial bullae, and progressive corneal clouding reducing visual acuity.[4–6] Early-stage disease is typically managed with topical hypertonic saline to reduce symptoms, such as corneal swelling, but surgical intervention is currently the only treatment option available to individuals with advanced disease to restore bilateral vision and prevent blindness.[6] Full or partial keratoplasty are invasive procedures that rely upon specialist facilities and the availability of healthy donor material, of which there is a global shortage.[7] These issues, coupled with the global aging population, highlight the need for alternative and effective treatment strategies.

In 2010, a landmark FECD genome-wide association study (GWAS) identified a strong association with common non-coding variants located within the transcription factor encoding gene *TCF4* (MIM: 602272). The risk allele of the most highly associated SNP (rs613872) conferred a remarkably high odds ratio (OR) of 5.5 for individuals carrying one copy or an OR of 30 for individuals with two copies.[8] Subsequently, the SNP rs613872 was found to be in linkage disequilibrium with CTG18.1, a CTG repeat expansion situated within an intronic region of *TCF4*, connecting the initial GWAS signal with a putative functional variant.[9] This association has now been replicated in a range of ethnically distinct cohorts, supporting the hypothesis that expanded copies of the CTG18.1 repeat are associated with FECD.[10–14]

Transcripts containing expanded copies of the CUG repeat accumulate as discrete nuclear RNA foci within tissue derived from individuals affected with FECD;[15] similar to other repeat expansion-associated disorders, such as myotonic dystrophy type 1 (DM1 [MIM: 160900]), which is caused by an identical CTG repeat expansion located within the 3′ untranslated region (UTR) of *DMPK* (MIM: 605377).[16,17] DM1 pathogenesis has largely been attributed to these RNA aggregates sequestering RNA-splicing

[1]UCL Institute of Ophthalmology, London ECIV 9EL, UK; [2]ProQR Therapeutics, Zernikedreef 9, 2333 CK Leiden, the Netherlands; [3]Research Unit for Rare Diseases, Department of Paediatrics and Adolescent Medicine, First Faculty of Medicine, Charles University and General University Hospital in Prague, Ke Karlovu 2, Prague 128 08, Czech Republic; [4]Department of Ophthalmology, First Faculty of Medicine, Charles University and General University Hospital in Prague, U nemocnice 2, Prague, Czech Republic; [5]Moorfields Eye Hospital, London EC1V 2PD, UK; [6]Institute of Aging and Chronic Disease, University of Liverpool, Liverpool L7 8TX, UK; [7]Department of Ophthalmology and Twin Research, King's College London, London SE1 7EH, UK
[8]These authors contributed equally to this work
[9]These authors contributed equally to this work
*Correspondence: alice.davidson@ucl.ac.uk
https://doi.org/10.1016/j.ajhg.2018.02.010.
© 2018 The Author(s). This is an open access article under the CC BY license (http://creativecommons.org/licenses/by/4.0/).

Table 1. Summary of CTG18.1 Genotyping Data in the FECD Cohort

	N	NE/NE	E/NE	E/E	≥1 E
Total FECD cohort (mean age = 69)	450	23.6% (106/450)	72.4% (326/450)	4.0% (18/450)	76.4% (344/450)
Females (mean age = 70)	265	27.2% (72/265)	69.1% (183/265)	3.8% (10/265)	72.8% (193/265)
Males (mean age = 68)	185	18.4% (34/185)	77.3% (143/185)	4.3% (8/185)	81.6% (151/185)
Subjects recruited at MEH	318	25.5% (81/318)	70.1% (223/318)	4.4% (14/318)	74.5% (237/318)
White (82.4%)	260	22.7% (59/260)	71.9% (187/260)	5.4% (14/260)	77.3% (201/260)
Other (17.6%)	58	37.9% (22/58)	62.1% (36/58)	0.0% (0/58)	62.1% (36/58)
Subjects recruited at GUH (white)	132	18.9% (25/132)	78.0% (103/132)	3.0% (4/132)	81.1% (107/132)
AMD cohort (mean age = 78)	550	95.8% (527/550)	4.2% (23/550)	0.0% (0/550)	4.2% (23/550)
Females (mean age = 78)	356	96.1% (342/356)	3.9% (14/356)	0.0% (0/356)	3.9% (14/356)
Males (mean age = 78)	194	95.4% (185/194)	4.6% (9/194)	0.0% (0/194)	4.6% (9/194)

Expanded alleles are defined as ≥50 CTG repeats. Abbreviations are as follows: NE, non-expanded CTG18.1 allele; E, expanded CTG18.1 allele; MEH, Moorfields Eye Hospital; GUH, General University Hospital in Prague.

factors, including MBNL1 and MBNL2,[18–20] leading to a functional deficiency of these proteins and subsequent global disruption of splicing.[21,22]

Studies using DM1 cell and animal models have shown that targeting the CTG expansion within *DMPK* using an antisense oligonucleotide (ASO) approach leads to a reduction in RNA foci and downstream markers of toxicity.[23,24] To determine whether similar strategies could be effective for CTG18.1-associated FECD and to translate FECD therapies into the clinic, appropriate FECD disease models are essential. Given the lack of animal models for FECD, coupled with the general poor association of animal model phenotypes with human complex disease, corneal endothelial cell (CEC) cultures derived from affected individuals offer an ideal opportunity to probe disease mechanism and investigate therapeutic approaches.

Here we demonstrate that the *TCF4* CTG18.1 expansion confers a highly significant disease risk in our large cohort of individuals affected by FECD. Our data define trinucleotide repeat size as a fundamental driver of RNA foci incidence in CECs. We identify downstream markers of RNA toxicity and assess the effectiveness of a targeted ASO treatment strategy for CTG18.1 expansion-associated FECD.

Material and Methods

Subject Recruitment and Phenotyping

The study followed the tenets of the Declaration of Helsinki and was approved by Moorfields Eye Hospital (MEH) ethics committee (REC reference 09/H0724/25) and the Ethics committee of the GUH, Czech Republic. Written informed consent was received from all participants included in this study. A total of 450 individuals (185 males and 265 females; mean cohort age, 69 years) were recruited to the study. Participants either had clinical signs of FECD (numerous corneal guttae on slit-lamp biomicroscopy) or had corneal transplantation surgery (either penetrating or endothelial keratoplasty) for FECD. The cohort was stratified based on gender and ethnicity (Table 1). For control purposes, DNA samples collected from 550 white European individuals with AMD were used in the study (194 males and 356 females; mean cohort age, 78 years) (Table 1). All risk calculations presented were performed using the white European samples only (392 FECD samples and 550 AMD samples).

TCF4 Expansion Genotyping

Genomic DNA was extracted from whole blood using conventional methodologies. A short tandem repeat (STR) assay was performed to genotype the CTG18.1 allele, in accordance with methods previously published by Wieben et al.[9] In brief, genomic DNA was amplified using a 5′FAM conjugated primer (5′-CAGATGAGTTTGGTGTAAGAT-3′) and an unlabeled reverse primer (5′-ACAAGCAGAAAGGGGGCTGCAA-3′). Post PCR product separation was performed on the ABI 3730 Electrophoresis 96 capillary DNA analyzer (Applied Biosystems). Data analysis was performed using GeneMarker software (SoftGenetics).

Collection of Endothelial Tissue Samples

Tissue derived from individuals affected by FECD was removed during endothelial keratoplasty surgery performed at MEH. As part of the procedure, 8 mm diameter discs of DM with attached endothelial cells were removed from the posterior surface of the central cornea. Control tissue, considered suitable for transplantation, was obtained from corneo-scleral rims stored in OptiSol-GS (Bausch & Lomb). All prepared tissue was stored in Lebovitz L15 Media (Life Technologies) supplemented with 1% antibiotic/antimycotic prior to being processed in the laboratory.

Primary CEC Culture

CECs retrieved from control tissue and from subjects diagnosed with FECD were cultured in accordance with a dual media approach described by Peh et al.[25] In brief, donated tissue comprising DM with attached endothelial cells was incubated in 0.2% collagenase type I powder in M5 media (Life Technologies) for 3 hr at 37°C to dislodge CECs from the DM. CECs were centrifuged and re-suspended again in M5 media to allow for cell adherence and stabilization. Media M5 contained Human Endothelial-SFM (Life Technologies) supplemented with 5% FBS,

1% antibiotic/antimycotic, and 0.1% selective ROCK inhibitor Y-27632 (AdooQ BioScience). Cells were seeded in cell culture ware pre-coated with FNC coating mixture (United States Biological). After 24 hr, to promote proliferation, culture media was replaced with M4 media containing Ham's F-12 Nutrient Mix GlutaMAX Supplement (Life Technologies)/Medium 199 GlutaMAX Supplement (Life Technologies), 20 μg/mL ascorbic acid, 1% insulin-transferrin-selenium (Life Technologies), 5% FBS, 1% antibiotic/antimycotic, 10 ng/mL bFGF (R&D Systems), and 0.1% selective ROCK inhibitor Y-27632 (AdooQ BioScience). Throughout culture, cells were kept in an incubator at 37°C, 5% CO_2 and medium was refreshed every 48 hr until the cells showed appropriate confluence for experimentation or passage. Cells were passaged a maximum of two times prior to any experiment being performed.

Generation of Fibroblast Cell Lines from Dermal Skin Biopsies

Primary fibroblast lines were generated as described by Carter et al.[26] Briefly, 5 mm skin biopsies were obtained under aseptic conditions and were shortly stored in DMEM/F-12, GlutaMAX (Life Technologies) supplemented with 10% FBS and 1% penicillin/streptomycin at 4°C until being processed. After the epidermal layer was removed, the biopsy samples were dissected into small pieces which were then immobilized under a glass coverslip for 24 hr (37°C, 5% CO_2). Fresh medium was added the following day and renewed every 2–3 days. At ~70% confluence, the cultures were passed through a cell strainer to remove pieces of tissue and finally seeded in T25 flasks.

Immunocytochemistry (ICC)

CECs grown on glass coverslips were fixed with 4% paraformaldehyde (PFA) in PBS for 10 min. After washing with PBS, cells were permeabilized with 0.1% Triton X-100 for 10 min and non-specific binding sites were blocked in PBS with 3% BSA and 10% donkey serum for 1 hr. The CECs were incubated with primary antibodies, diluted to the appropriate concentration (Table S1), in blocking solution overnight at 4°C. For MBNL1[27] and MBNL2 antibodies, 0.5% Triton X-100 was incorporated in the blocking solution instead, omitting the previous permeabilization step, and proceeding normally after. After washing with PBS, CECs were incubated in donkey fluorophore-bound secondary antibodies (Alexa Fluor 488, anti-mouse or anti-rabbit; Invitrogen) diluted 1:1,000 in 3% BSA PBS for 1 hr at room temperature. Cells were washed again and incubated in DAPI stain (1:5,000 dilution; Sigma) for 2 min. Finally, coverslips were mounted onto microscope slides using Fluorescent Mounting Medium (Dako). Appropriate negative controls were carried out by performing the same protocol without the addition of primary antibodies. Images were taken using a confocal Zeiss 700 microscope and processed with the Zeiss software.

ASO Transfections

CECs were transfected with ASOs complexed with DarmaFECT 4 transfection reagent (Dharmacon), in accordance with the manufacturer's instructions. The following ASOs were used; Control ASO: 5′-mG*mG*mU*mG*mG*mA*mU*mC*mA*mC*mG*mA*mG*mU*mU*mC*mA-3′, $(CAG)_7$ therapeutic oligo: 5′-mC*mA*mG*mC*mA*mG*mC*mA*mG*mC*mA*mG*mC*mA*mG*mC*mA*mG*mC*mA*mG-3′. m denotes 2′-O-Methyl ribonucleotide, * denotes phosphthioate linkages.

A final oligo concentration of 200 nM was selected for all experiments based on optimization data presented in Figure S1. All ASO treatment experiments were performed 24 hr post transfection.

Fluorescence *In Situ* Hybridization (FISH)

CECs grown in chamber slides were washed once with PBS and fixed with 4% PFA in PBS for 10 min at room temperature. Once fixed, cells were washed twice with PBS and permeabilized with 70% ethanol for 15 min at room temperature. Cells were rehydrated using a 50% formamide and 2 × SSC buffer for 5 min at room temperature. Cells were incubated overnight at 37°C in hybridization solution containing 50% formamide, 2 × SSC, 10% dextran sulfate, 0.2% BSA, 1 mg/mL yeast tRNA, and 12 μg/mL of Cy3-$(CAG)_7$ probe. Cells were washed thoroughly using 50% formamide in 2 × SSC followed by 50% formamide in 0.1 × SSC before being stained with DAPI (1:5,000 dilution; Sigma) for 2 min at room temperature. Cells were washed with PBS and coverslips were mounted onto the microscope slide using fluorescent mounting medium (Dako). Images of foci were taken using a confocal Zeiss 700 microscope and processed with the Zeiss software.

Quantitative Foci Image Analysis

Z stack images of transfected and stained CECs were processed using Zeiss software. Images for each independent CEC line were taken using the same image acquisition parameters. Post capture, z stacks were processed as maximum intensity projection images. RNA foci were quantified by segmentation using CellProfiler (Broad Institute).[28] Nuclei were defined in the 405 nm (DAPI) channel, after a median filter was applied, by adaptive maximum correlation thresholding, followed by form-factor and eccentricity filtering. RNA foci were defined in the 561 nm (Cy3) channel, after a median filter was applied, as objects within the perimeter of a nucleus, using per-nucleus robust background thresholding, with parameters set per cell line, due to differences in staining intensity. Cells with high background in FISH staining, defined as mean intensity per nucleus, were discarded to reduce segmentation error. A minimum of 100 nuclei per independent condition and cell line were analyzed.

Dual FISH and ICC Assay

Following PFA fixation, cells were washed with PBS and then incubated in blocking solution containing 10% donkey serum, 0.5% Triton X-100, 3% BSA, and 0.4 U/μL RNase inhibitor in PBS for 1 hr at room temperature. Cells were subsequently incubated with mouse anti-MBNL1 antibody (1:1,000 in blocking solution; kindly provided by Thornton Lab, Chicago) overnight at 4°C. Following primary antibody incubation, cells were washed with PBS and incubated with secondary anti-mouse antibody conjugated with Alexa Fluor 488 (1:1,000 in 0.5% Triton X-100, 3% BSA) for 1 hr at room temperature. Cells were again washed with PBS and post-fixed in 4% PFA in PBS for 10 min. FISH was subsequently performed, in accordance with methods stated above.

Quantitative Image Analysis of MBNL1 Puncta

A minimum of ten images per condition for each transfected and stained CEC line were taken for analysis purposes, using a confocal Zeiss 700 microscope. In the same way as previously described, CellProfiler software was used to identify and quantify MBNL1 puncta, defined in the Cy2 channel. The percentage of objects found in a nucleus after treatment with both ASOs was plotted individually, per cell line investigated (Figure S2).

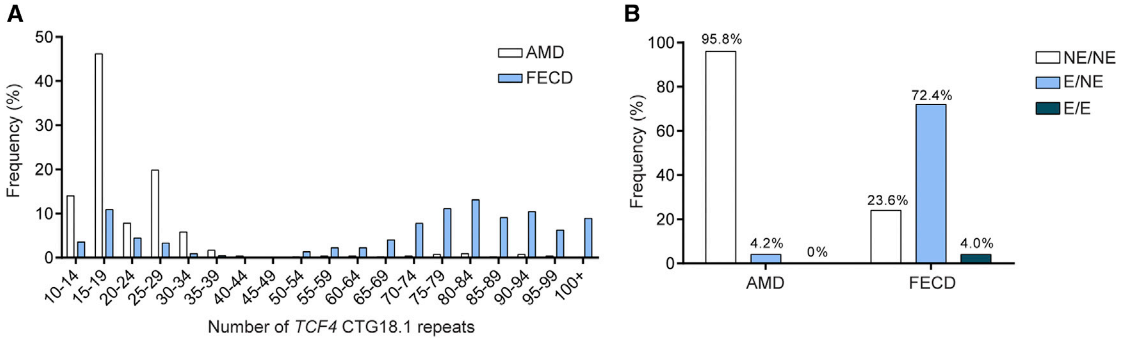

Figure 1. Expansion of CTG18.1 Is Associated with FECD in a British and Czech Cohort
(A) Frequency histogram comparing relative distribution of CTG repeat length in Fuchs endothelial corneal dystrophy (FECD) and age-related macular degeneration (AMD) cohorts. The longest allele detected, per individual tested, is shown. In total the FECD (blue) and AMD (white) cohorts comprised 450 and 550 individuals, respectively.
(B) Bar chart illustrating the relative frequency of individuals with both alleles non-expanded (NE/NE), one expanded allele (E/NE), or both alleles expanded (E/E) in both the FECD and AMD cohorts. Expanded alleles are defined as ≥50 CTG repeats.

Analysis of Pre-mRNA Splicing

Total RNA was extracted from primary CECs using NucleoSpin RNA XS kit according to manufacturer's guidelines (Macherey-Nagel). cDNA was reverse-transcribed using a Tetro cDNA synthesis kit (BIOLINE) with an oligo $(dT)_{18}$ primer mix in accordance with manufacturer's guidelines. Reverse transcription PCR was performed using intron spanning primers listed in Table S2 using GoTaq Green Master mix (Promega) and standard cycling parameters. The identities of all amplified products were confirmed by Sanger sequencing using standard methodologies. The relative intensities of PCR-amplified products resolved on agarose gels were calculated using Image Lab software package (BioRad).

Intraocular Administration of ASOs

C57BL/6 mice (n = 12) were given a single intravitreal administration into both eyes of Cy-3-labeled 2′Ome-PS-$(CAG)_7$ at doses of 0.025, 0.01, or 0.05 mg in 1 μL of PBS, PBS only, or a molar equivalent of free Cy3 label conjugated to the ASO. Mice were sacrificed at 48 hr following intravitreal injection (IVT). IVT injection method: Mice were anesthetized using ketamine hydrochloride/medetomidine hydrochloride i.p. Both eyes were dilated with 1% tropicamide and 2.5% phenylephrine drops. Injections of 1 μL were made below the limbus using a 32 g needle attached to a 2.5 μL Hamilton syringe, and animals were then recovered using antisedan.

Immunohistochemistry

Eyes were enucleated and immersion fixed in 4% PFA for 24 hr prior to rapid freezing in OCT imbedding compound. Eyes were removed and cryoprotected by overnight incubation (at 4°C) in 30% sucrose solution. Corneal tissue sections (16 μm) were cut on a cryostat and collected onto charged slides and viewed immediately on a confocal fluorescence microscope.

Statistical Analyses

To test for associations between FECD disease affection status and polymorphic markers, we built logistic regression models in the above described groups of affected subjects and control subjects. FECD diagnosis was the outcome and the number of alleles with 50 or more repeats was used as the independent variable. Three association models were built: the first model was run in both male and female participants and included adjustment for sex. The other two models were sex specific, restricted to strata of male and female participants only (therefore not adjusted for sex). All the models were tested for significance using the "glm" function from the R 3.4.1 statistical software base packages.

Statistical analyses of CECs were performed with GraphPad Prism 6 software. A chi-squared test was used to analyze differences in the distribution of foci incidence between the cells treated with the control and the $(CAG)_7$ ASO. Odds ratio tests were performed to analyze whether the likelihood of finding 0, 1, 2, 3, or 4 or more foci after treatment was increased or decreased. An unpaired two-tailed t test was used to calculate the difference in mean number of MBNL1-positive foci per nucleus between control and $(CAG)_7$ ASO treatments. A one-way analysis of variance (ANOVA) using Dunnett's multiple comparisons test with a single pooled variance was used to analyze differences between mean amplicon expression in expansion-negative and expansion-positive CEC lines compared with controls. Paired two-tailed t tests were conducted to analyze the effect of $(CAG)_7$ versus control ASO treatment on amplicon expression, for each respective transcript investigated. Data are represented as means ± 1 SD for bar graphs throughout the manuscript.

Results

Expansion of the CTG18.1 Trinucleotide Repeat Confers Significant Risk for FECD

A highly significant association between expansion of the CTG18.1 trinucleotide repeat (conservatively defined as ≥50 repeats) and FECD was identified (OR = 76.47; 95% CI: 47.45–123.2; $p = 5.69 \times 10^{-71}$) in the white European-only portion of the cohort (n = 392; Table 1). The distribution of the CTG18.1 expansion lengths among individuals affected by FECD and age-related macular degeneration (AMD), used as an ethnically matched control population for the purpose of this study, are summarized in Figures 1A and 1B and Table 1. For the AMD cohort, 4.2% (23/550) had one expanded copy (≥50 repeats) of the CTG18.1 allele, in line with reports from other unaffected populations screened for control purposes,[9,12–14] and none were found to have two expanded alleles. In contrast, 76.4% (344/450) of the FECD cohort

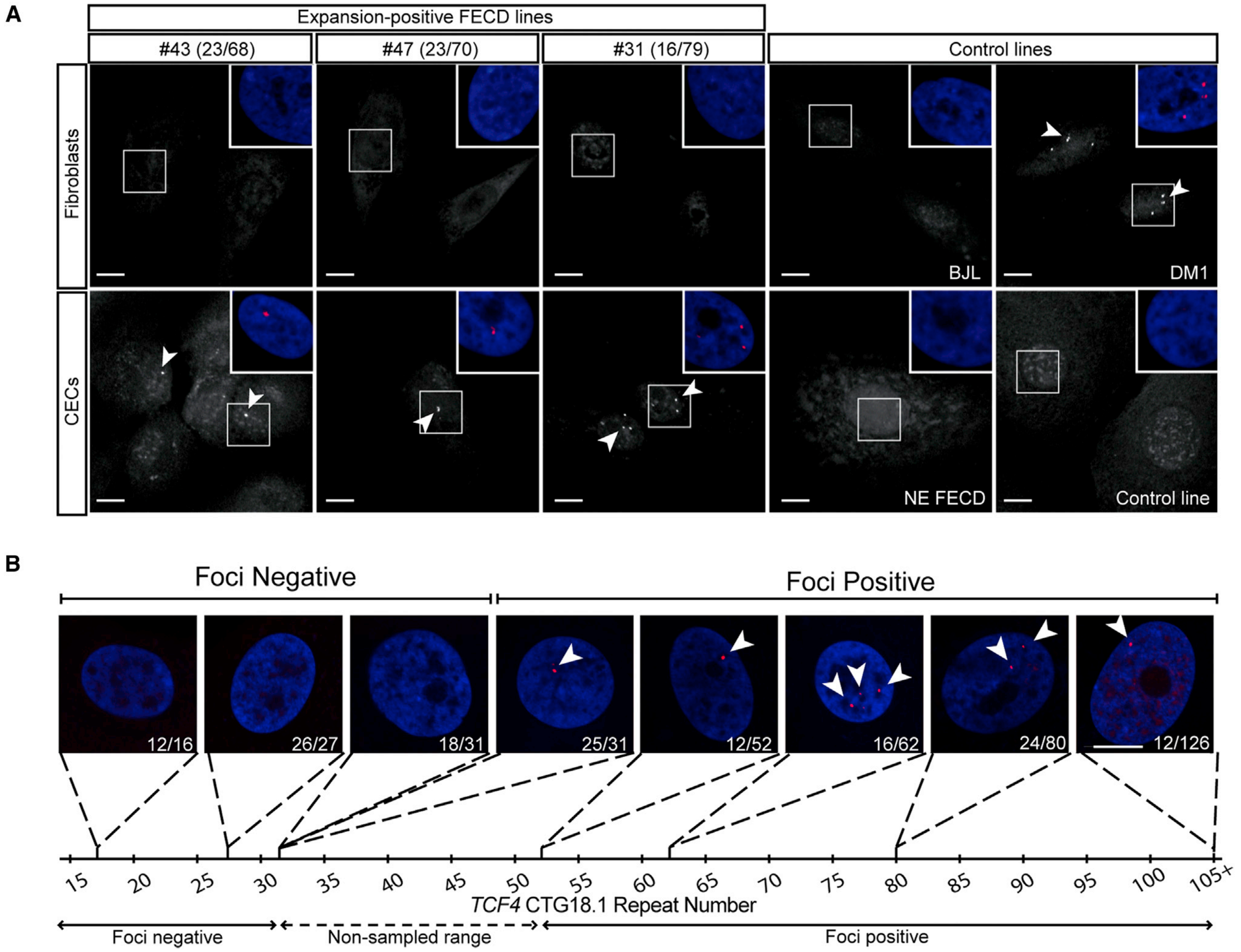

Figure 2. CTG18.1-Associated RNA Foci Occur in a Tissue-Specific Manner
(A) Fluorescence *in situ* hybridization (FISH) was used to detect CUG-specific RNA foci in fibroblast and corneal endothelial cells (CECs) lines derived from three individuals with FECD and expanded *TCF4* alleles. Fibroblast line BJL, non-expanded (NE) FECD, and healthy CECs were used as negative controls. Myotonic dystrophy 1 (DM1) fibroblasts were used as a positive control for foci detection (arrowheads). Each image is presented in greyscale and foci are indicated with arrowheads. Color insets (zoom panels) are presented.
(B) Representative images of foci incidence among CECs derived from FECD-affected subjects with increasing CTG18.1 repeat lengths. Nuclei are stained with DAPI (blue). Foci detection was performed using Cy3-(CAG)$_7$ probe (red, arrowheads). Scale bars, 10 mm.

had one or more expanded copies of the CTG18.1 allele, of which 4.0% (18/450) had bi-allelic expansions. Interestingly, male subjects had a higher incidence of expanded CTG18.1 alleles (81.6% versus 72.8% with at least one expanded allele; Table 1) and the FECD risk associated with repeat expansion at this locus was higher in males (OR = 95.04, 95% CI: 43.08–209.70, $p = 1.62 \times 10^{-29}$) than in females (OR = 66.78, 95% CI: 36.79–121.20, $p = 2.06 \times 10^{-43}$), supporting the hypothesis that interaction of this locus with gender could be important.[29]

CEC Cultures as a Model of FECD

To investigate CTG18.1 expansion-associated pathology, the occurrence of stable sense-strand-derived CUG RNA foci in fibroblast primary cultures was investigated in six independent fibroblast lines (F#1–6) derived from FECD-affected subjects with expanded CTG18.1 genotypes (Table S3). For each line, FISH was performed using a Cy3-(CAG)$_7$ probe to determine the incidence of RNA-specific foci (Figure S3). Despite identifying multiple bright nuclear foci in a fibroblast line derived from a DM1 subject (positive control), none were detected in any of the FECD fibroblast lines investigated (Figure 2A).

On this basis, we tested the potential of using primary CECs, derived from tissue excised during endothelial keratoplasty, to investigate CTG18.1 expansion-associated pathology. The native "endothelial-like" properties of the CECs were confirmed by ICC and a variety of endothelial markers including sodium-potassium transporting adenosine triphosphatase (ATP1A1), zonula occludens 1 (ZO-1), N-cadherin, N-CAM, and CD166[30–32] (Figure S4). Furthermore, the cultured CECs displayed distinctive polygonal morphology.[25,33,34] FISH was performed, using the same Cy3-(CAG)$_7$ probe as described above, in three distinct

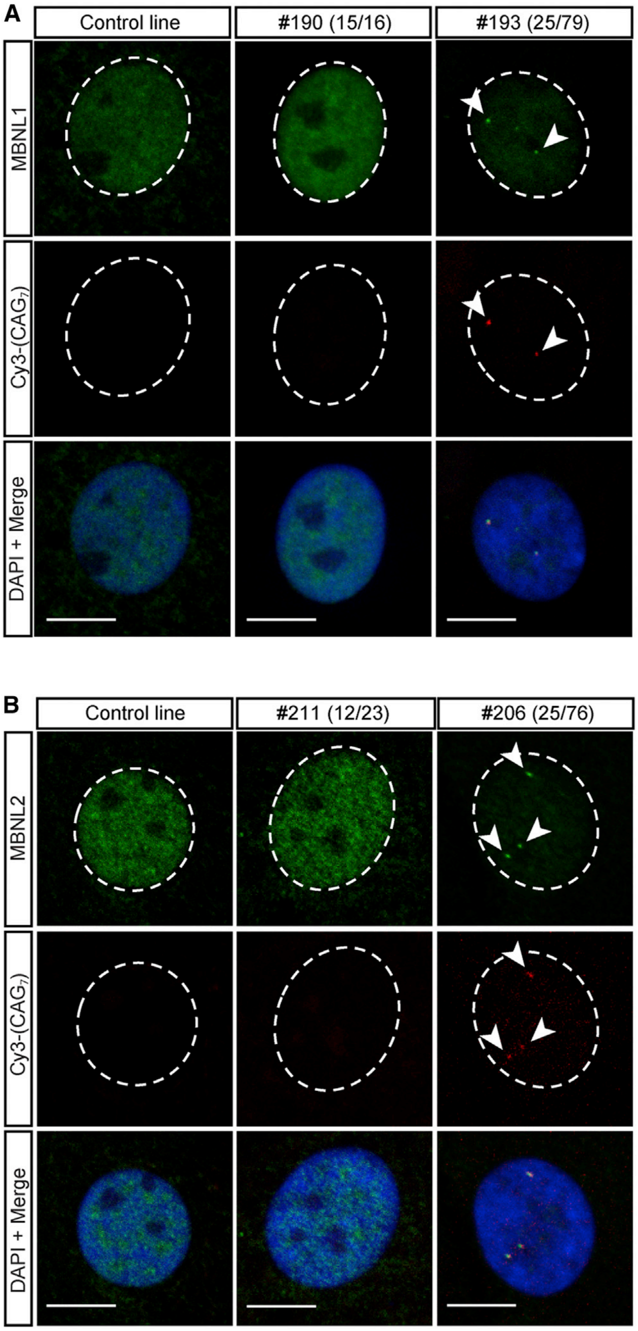

Figure 3. MBNL1 and MBNL2 Are Sequestered to RNA Foci in Corneal Endothelial Cells (CECs) Derived from FECD-Affected Subjects with CTG18.1 Expansions

Representative images of MBNL1 (A) and MBNL2 (B) protein nuclear localization in cell lines derived from expansion-positive FECD-affected subjects, expansion-negative FECD-affected subjects, and CECs derived from healthy individuals. RNA foci are labeled with a Cy3-$(CAG)_7$ FISH probe and DAPI is used to stain nuclei. Co-localization of the MBNL proteins and RNA foci is represented in the bottom row of both panels. Scale bars, 10 μm.

primary FECD CEC lines and corresponding individual-matched fibroblast lines (Figure 2A). In each instance, bright nuclear foci were detected in the FECD CECs, similar to those previously identified in corneal tissue,[15,35] whereas the corresponding fibroblasts were foci negative, suggesting that the endothelial-specific context is important and that the cultured primary CECs represent an ideal *ex vivo* system to investigate CTG18.1 expansion-associated corneal endothelial pathology (Figure 2A).

RNA Foci Are a Biomarker of CTG18.1-Associated Pathology in CECs

To further explore the incidence of RNA foci, we investigated a total of 36 independent CEC lines derived from FECD-affected subjects by FISH (Table S4; Figure 2B). In summary, no foci were detected in 9 CEC lines derived from FECD-affected subjects with CTG18.1 genotype status ranging from 12/12 to 18/31, in addition to a further 4 control lines. Bright nuclear foci were clearly detected in 27 CEC lines derived from individuals with alleles ranging from 25/31 to 12/126. Interestingly, CTG18.1 allele length of 31 repeats appears to represent a critical threshold for foci occurrence in CECs as individuals with genotypes of 18/31 and 25/31 were foci negative and positive, respectively. No samples were available with an expansion in the range between 32 and 52 repeats (Figure 2B; Table S4). On this basis, we classified CEC lines selected for further experimental investigation as non-expanded (NE) if both CTG alleles contained <31 repeats, whereas those with at least one allele ≥53 repeats were considered expanded (Tables S3–S9).

RNA Splicing Factors MBNL1 and MBNL2 Are Sequestered by Nuclear RNA Foci

To determine whether RNA binding proteins were being sequestered by the RNA foci, in the cultured FECD CECs, we employed a dual FISH and ICC approach. The nuclear distribution of MBNL1 and MBNL2 was investigated in multiple CEC lines derived from individuals with and without expanded copies of the repeat (Table S5). MBNL1 displayed a diffuse nuclear localization in three non-expanded (NE) CEC lines and no foci were detected, as anticipated (Figure 3A). Striking co-localization was observed between MBNL1 and the CUG-specific RNA foci in all three independent CTG18.1 expansion-positive lines examined (Figure 3A), concordant with a previous observation made in FECD-diseased tissue.[15] Similarly, the recruitment of MBNL2 to CUG-specific nuclear RNA foci was also detected in expansion-positive CECs (n = 3) and MBNL2 displayed diffuse nuclear localization in expansion-negative and control CEC lines (n = 4) (Figure 3B). These data demonstrate that RNA splicing factors are recruited to CUG RNA foci and suggest that this recruitment may induce a functional deficiency of proteins that would be predicted to alter alternative splicing events, in a similar downstream pathway to what has been established for DM1.[18–22,36]

Expansion of CTG18.1 Is Associated with Altered mRNA Processing

Transcriptomic analysis of endothelial tissue derived from individuals affected with FECD has previously suggested

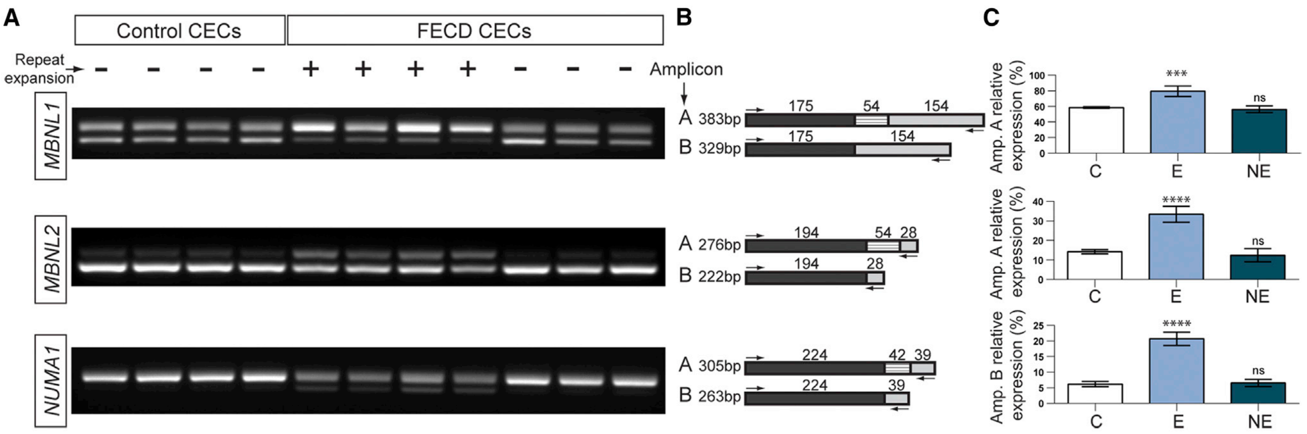

Figure 4. Altered Pre-mRNA Splicing Events Are Specific to CTG18.1 Expanded Corneal Endothelial Cells (CECs) Derived from FECD-Affected Subjects

(A) Reverse transcriptase (RT)-PCRs reactions are shown for three selected alternative splicing events investigated for the following transcripts: *MBNL1*, *MBNL2*, and *NUMA1*. Samples are grouped in the following categories; controls (lanes 1–4), FECD CTG18.1 expansion positive (lanes 5–8), and FECD CTG18.1 expansion negative (lanes 9–11).

(B) Schematic representations of RT-PCR-generated amplicons are provided for each transcript-specific reaction. Primer locations are denoted with arrows. The respective sizes of all amplified products are given.

(C) Percentage expression of amplicons of interest (A or B) relative to total amplified products, per reaction, are presented as a mean for each respective group (C, E, and NE). Error bars represent ± 1 standard deviation. p values were calculated by one-way analysis of variance (ANOVA); ****$p < 0.0001$, ***$p < 0.001$, ns, non-significant.

abnormal regulation of alternative pre-mRNA splicing in CTG18.1 expansion-positive tissue.[15,37] Therefore, we investigated whether signatures of differential splicing were present in cultured primary CECs. Total RNA was isolated from four FECD CTG18.1 expansion-positive and three FECD CTG18.1 expansion-negative CEC lines (CTG18.1 genotype listed in Table S6), in addition to four CEC lines derived from healthy control subjects. RT-PCR analysis was performed to investigate differential splicing of the three most robustly detected aberrant events observed in tissue derived from affected subjects: *MBNL1* (MIM: 606516), *MBNL2* (MIM: 607327), and *NUMA1* (MIM: 164009).[37] For each transcript analyzed, significantly different ($p < 0.001$) patterns of splicing were observed only in the FECD expansion-positive lines compared to FECD expansion-negative and unaffected control lines, supporting the hypothesis that abnormal regulation of mRNA processing is specific to CTG18.1-related pathology (Figure 4).

ASO Treatment Reduces the Incidence of Nuclear RNA Foci

A fully 2′-O-methyl-phosohorothioate(2′-O-Me-PS) modified $(CAG)_7$ ASO complimentary to $(CUG)_n$ repeats has previously been shown to effectively silence *DMPK1* trinucleotide repeat expansion transcripts in a DM1 humanized animal model and human DM1 cell system and to reduce the number of DM1-associated RNA foci in a repeat-length-dependent manner.[23,24] We therefore tested whether transfecting with 2′-O-Me-PS-$(CAG)_7$ ASOs, complimentary to *TCF4* CTG18.1-derived transcripts, could induce a similar reduction in CUG-specific RNA foci, a biomarker of CTG18.1-related pathology, in the CEC cultures.

A series of six FECD CEC lines, with confirmed expanded CTG18.1 genotypes (Table S7), were selected for ASO treatment. Each independent line was transfected with 200 nM of either $(CAG)_7$ or a control ASO of identical chemical structure and of comparable length but specific to a completely unrelated sequence. A final oligo concentration of 200 nM was selected for all experiments based on optimization data (Figure S1). All lines analyzed showed a striking reduction in foci number in response to $(CAG)_7$ ASO treatment (Figures 5 and S5). Violin plots summarize the shift in distribution of RNA foci-positive nuclei observed comparing the control ASO-treated CECs versus $(CAG)_7$ ASO treatment ($n = 6$) (chi square test, $\chi^2 = 160.78$, $df = 4$, $p = 0.001$; Figure 5B). The percentage of nuclei containing zero, one, two, three, and four or more foci was analyzed, and odds ratio (OR) test confirmed that the likelihood of finding zero foci was significantly increased in cells treated with the $(CAG)_7$ ASO compared to cells treated with the control ASO ($OR = 6.2024$, 95% CI, $p < 0.0001$).

ASO Treatment Rescues MBNL1 Nuclear Localization

To determine the effect of $(CAG)_7$ ASO treatment on MBNL1 distribution, four independent expansion-positive CEC lines (Table S8) were selected and ICC was performed, following treatment with either the $(CAG)_7$ or control ASO. In all four lines treated with the $(CAG)_7$ ASO, there was an obvious redistribution of MBNL1 and a significant reduction in MBNL1-positive puncta ($p < 0.0001$), demonstrating that ASOs can rescue aberrant MBNL1 nuclear localization associated with the CTG18.1 expansion-related pathology (Figures 3A, 5D, 5E, and S2).

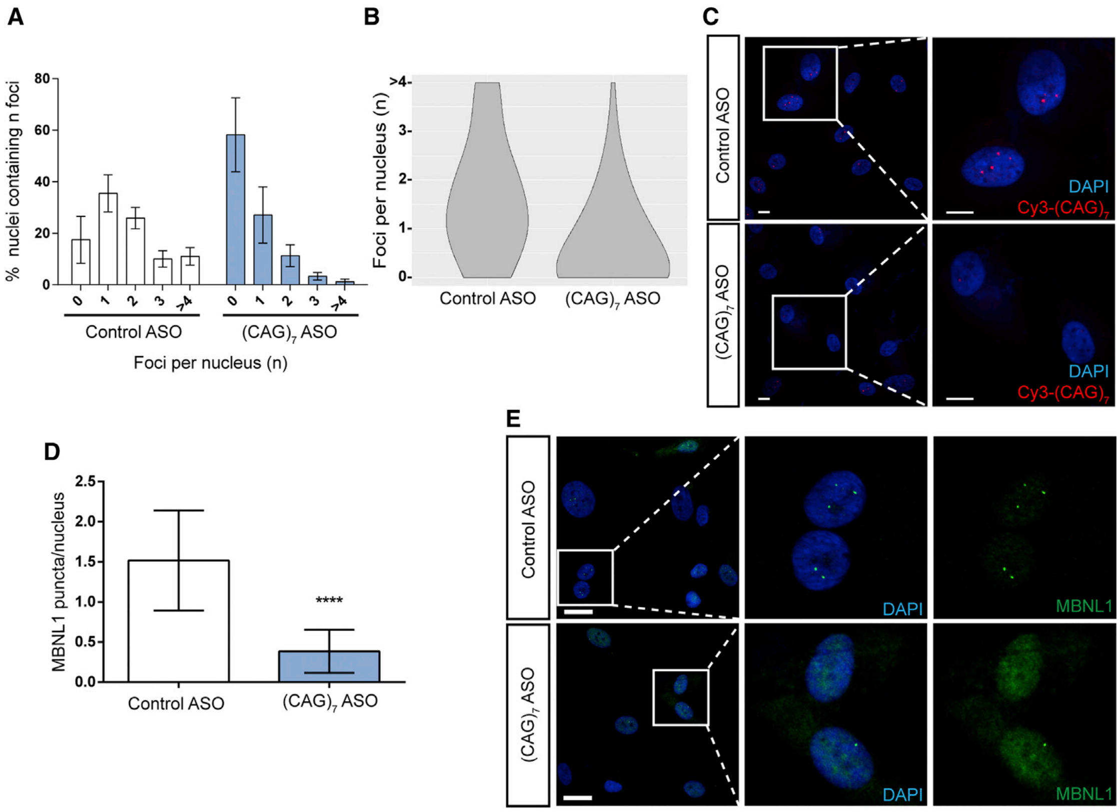

Figure 5. ASO-Mediated Treatment of Corneal Endothelial Cells (CECs) Significantly Reduces Foci Number and Rescues MBNL1 Nuclear Localization

(A) Foci incidence for control and $(CAG)_7$ antisense oligonucleotide (ASO)-treated FECD-affected subject-derived CECs. The graph shows percentages of nuclei that contain 0, 1, 2, 3, and 4 or more foci after treatment with the different ASOs. Mean ± SD are represented in each case (n = 6).

(B) Violin plots representing the distribution of the frequencies of each group of nuclei (containing 0, 1, 2, 3, and 4 or more foci) in cells treated with control and $(CAG)_7$ ASOs (n = 6).

(C) Representative images of ASO treatment on foci incidence. Sense RNA foci detection using Cy3-$(CAG)_7$ probe (red). Scale bars, 10 μm.

(D) Number of MBNL1 puncta present per nucleus when cells were treated with either a control ASO or with the $(CAG)_7$ ASO. The mean ± SD from 4 independent expansion-positive CEC lines, where a minimum of 95 nuclei were evaluated per line. p values were calculated using an unpaired two tailed t test; ****$p < 0.0001$.

(E) Representative example illustrating the reduction of MBNL1 puncta and the changes in MBNL1 localization when cells were treated with control and $(CAG)_7$ ASOs. In all cases CECs were treated with 200 nM $(CAG)_7$ or control ASO for 24 hr. Scale bars, 25 μm.

ASO Treatment Reduces Aberrant mRNA Processing

We investigated the potential of $(CAG)_7$ ASO treatment to reverse the shift in alternative splicing events previously observed in expansion-positive CEC lines (Figure 4). Ten FECD expansion-positive CEC lines (Table S9) were treated with either the $(CAG)_7$ or control ASO. Following treatment, RNA was extracted and RT-PCR was performed. For both *MBNL1* and *MBNL2* there was a highly significant ($p < 0.0001$) shift in the relative proportions of alternatively spliced transcripts after $(CAG)_7$ ASO treatment (n = 10) toward the control CEC spliceoform distribution (Figure 6). Furthermore, a significant ($p \leq 0.05$) shift in the relative proportions of alternatively spliced transcripts was also demonstrated for *NUMA1* (n = 10) (Figure 6). These data therefore demonstrate that the $(CAG)_7$ ASO is effective at rescuing the differential splicing events associated with CTG18.1 expansion-related pathology (Figures 4 and 6).

Intraocular Injections Enable Effective *In Vivo* Delivery of ASOs to the Corneal Endothelium

To assess the likely accessibility of ASOs to the corneal endothelium, C57B16 mice were injected intravitreally with varying concentrations (0.025, 0.01, and 0.005 mg) of Cy-3-labeled 2′Ome-PS-$(CAG)_7$. Confocal fluorescence microscopy revealed that the ASO was present in corneal endothelium, keratocytes, and stroma, specifically accumulating in both the nuclear and perinuclear region of both the endothelial and stromal cells. This localization increased in a dose-dependent manner, showing stronger accumulation in cell layers at 48 hr post-dosing (Figure S6). ASOs with identical chemistry, following intraocular injection, have previously been shown *in vivo* to display a similar peri-nuclear and nuclear localization in other cell types in conjunction with effective molecular activity.[38] These data highlight the potential for the $(CAG)_7$ ASO to target CECs *in vivo*, an essential prerequisite for the effective delivery of an ASO-mediated FECD therapy.

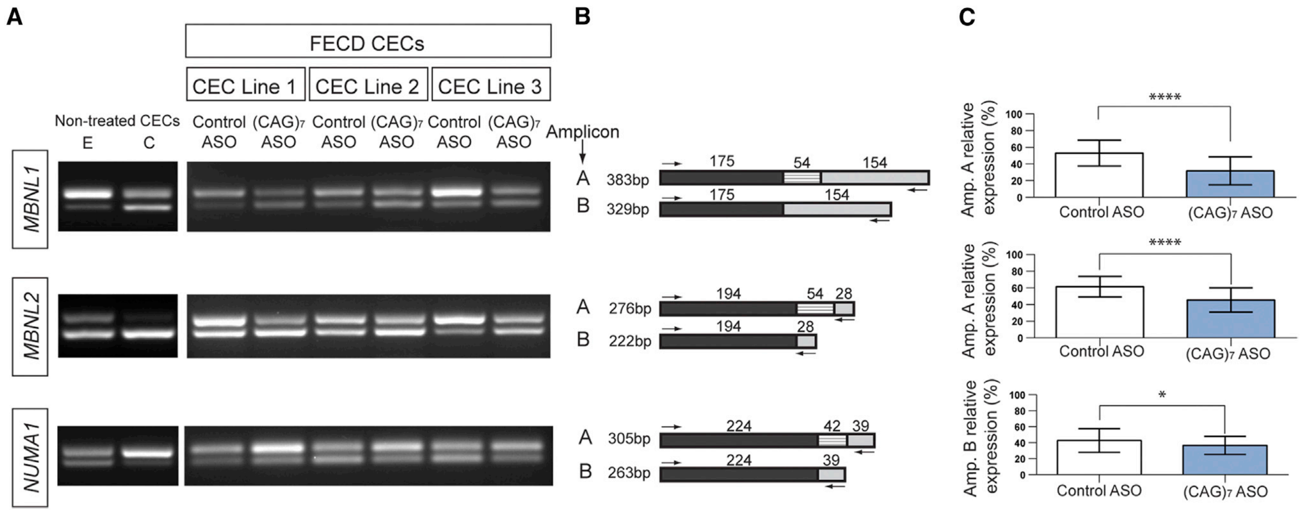

Figure 6. ASO Treatment Rescues Differential Splicing Events Underlying CTG18.1-Associated Pathology
(A) Representative RT-PCR images for non-treated samples with either expanded (E) or control (C) (non-expanded CTG18.1 genotypes) are shown on the far left, for reference purposes. Representative RT-PCRs are shown for three independent ASO-treated CEC lines.
(B) Schematic representations are provided for each respective reaction. Primer locations are denoted with arrows. The respective sizes of all amplified products are given.
(C) Percentage expression of amplicons of interest (A or B) relative to total amplified products, per reaction, are presented as a mean for control-ASO versus $(CAG)_7$ ASO-treated groups. The mean from 10 independent CEC lines ± SD. In all cases CECs were treated with 200 nM $(CAG)_7$ or control ASO for 24 hr. p values were calculated using a paired two tailed t test for each event investigated; ****$p < 0.0001$, *$p < 0.05$.

Discussion

ASOs have recently emerged as a powerful therapeutic option for disease intervention, including those caused by trinucleotide repeat expansions.[10,39–42] Here we show that a non-coding CTG trinucleotide repeat expansion in *TCF4* (CTG18.1) confers greater than 76-fold risk for FECD in a large white British and Czech cohort. We demonstrate that primary CECs derived from FECD-affected subjects display the predicted hallmarks of primary and downstream repeat-expansion-associated pathology, and subsequently show that these changes are reversed by an ASO treatment specifically targeted at the CTG18.1 trinucleotide repeat expansion. An ASO-based treatment could therefore offer an innovative therapeutic approach that could benefit a substantial number of individuals affected by this common and sight-threatening condition.[43]

The data presented here suggest that the *TCF4* repeat expansion leads to CEC-specific dysfunction, as unlike other trinucleotide expansion diseases, nuclear RNA foci are not observed in case-matched fibroblasts. These data, at least in part, explain the corneal-specific phenotype resulting from repeat expansions in this widely expressed gene and highlight the importance of investigating the trinucleotide expansion in primary human CECs. *TCF4* haploinsufficiency causes the systemic condition Pitt-Hopkins syndrome, but the noncoding repeat expansion exclusively affects the cornea.[44,45] Interestingly, a recent study has reported that FECD is a common ocular finding in DM1-affected case subjects.[46] Combined with our data, this suggests that the corneal endothelium is susceptible to toxicity induced by these genetically distinct repeat expansions, driven by RNA foci.

It is well established for a wide range of repeat expansion disorders that disease onset and incidence of RNA foci manifest only above a critical level of nucleotide repeats.[47] A threshold for CTG18.1 repeat length and FECD association is yet to be fully defined. Performing FISH with 36 distinct CEC lines derived from FECD-affected subjects has enabled us to identify the threshold for the number of repeats required to produce nuclear RNA foci in our model (Figure 2B; Table S4). Nine CEC lines with CTG18.1 genotypes ranging from 12/12 to 18/31 were found to lack RNA foci. A further 27 lines with CTG18.1 genotypes ranging from 25/31 to 12/126 were all found to exhibit punctate nuclear RNA foci. These data allow us to correlate CTG18.1 genotype status with CUG RNA foci incidence and indicate that a repeat size of more than 31 trinucleotide repeats is sufficient to drive the accumulation of stable CUG RNA foci in primary CECs (Figure 2B; Table S4). This identified threshold also correlates notably with the binominal distribution of CTG18.1 repeat length observed in our FECD cohort (Figure 1A), which is likely attributed to the instability of the repeat above approximately 30 copies. Interestingly, RNAs containing more than 30 CUG repeats have recently been demonstrated to undergo phase separation to form nuclear foci.[47] The threshold for this repeat length-dependent process (30 CUG repeats) is remarkably similar to what we have observed with respect to CTG18.1-related foci occurrence in CECs (Figure 2B). These phase separation data further support the use of agents that disrupt RNA-RNA base-pairing, such as ASOs,

as viable treatment options for RNA foci-induced cellular toxicity.[47] Taken together, these data suggest that a CTG18.1 length $\geq$32 should, in future, be considered as FECD risk associated. We repeated the tests for association with FECD defining the expanded repeats as $\geq$32, instead of the previously used more conservative threshold of $\geq$50. The association model became more significant ($p = 3.79 \times 10^{-74}$), although the disease risk conferred by this locus was lower (OR = 34.14; 95% CI: 23.35–49.91). Future analysis of CECs from individuals affected by FECD with repeat lengths in the unidentified range of 31–53 could further refine this important threshold.

We investigated the downstream consequences of RNA foci and have identified that sequestration of RNA splicing factors, MBNL1 and MBNL2 (Figure 3), in addition to abnormal patterns of alternative splicing were detectable in a repeat expansion-specific manner (Figure 4). These observations reinforce the notion that such RNA structures induce toxic gain-of-function effects that are likely to be disrupting overall cellular homeostasis, implicating aberrant RNA metabolism in the pathogenesis of CTG18.1-associated FECD.[15,37] Furthermore, these data demonstrate that these events are specific to CTG18.1-mediated FECD and are not a general downstream consequence of the disease, given that CECs derived from FECD-affected case subjects without expanded copies of the repeat did not display features of aberrant RNA metabolism.

Importantly, we demonstrate here that an ASO targeted to the CTG trinucleotide *TCF4* expansion can ameliorate disease-associated markers of RNA toxicity in CECs derived from FECD-affected subjects, specifically reducing RNA foci formation (Figures 5A–5C), prompting MBNL1 nuclear redistribution (Figures 5D and 5E) and partially suppressing differential splicing events (Figure 6). We additionally demonstrate that the ASO can access the corneal endothelium when injected intravitreally in mice (Figure S6). Entry of fluorescently labeled ASO to the corneal endothelial cells is an endogenous property of naked 2′Ome-PS-ASOs oligo and no other excipients are required to induce entry into these cells. Access to the CECs is also both dose and time dependent, suggesting that ASOs are rapidly taken up by cells after dosing. While it may be desirable to consider a topical eye drop therapy for FECD using an ASO approach, this is highly unlikely to be effective using naked ASOs given the structure of the corneal epithelium and the size and charge of ASOs; an adjunctive delivery technology would likely have to be considered in this case.

Future studies using CECs from affected individuals will be helpful in defining an effective ASO concentration, which can lead to a good clinical dose estimate when considering introduction to the anterior ocular chamber, which has a small fixed but rapidly exchanging volume. Detailed pharmacokinetic studies will be required to define the optimal dosing time interval, which is expected to be months, based on the rapid cellular uptake and the tissue/cellular longevity of similar 2′Ome-PS-ASOs in studies that have examined intraocular dosing of ASOs.[48] It is important to note that in the final stages of FECD there is significant endothelial cell loss, and consequently ASO-specific treatment is intended to prevent further cell loss. The most likely group of affected individuals to benefit from such a therapeutic intervention will be those individuals who are in the early stages of disease and have not yet experienced significant endothelial cell loss. Importantly, these at-risk individuals can be effectively identified by a CTG18.1 genotyping test.

In summary, we demonstrate proof-of-concept data that a targeted $(CAG)_7$ ASO treatment reduces gain-of-function RNA toxicity induced by *TCF4* CTG18.1 expansion, in a cellular and human genomic context. With the absence of FECD animal models, human *ex vivo* models are vital, both to provide a validation of the therapeutic approach for FECD and to continue the translation of ASO therapies into the clinic. ASO therapies are already in clinical trials for a variety of repeat expansion disorders including DM1[41] and Huntington disease (MIM: 143100), and additionally, intraocular ASO therapies are proposed for retinitis pigmentosa (RP [MIM: 268000]), RP associated with Usher syndrome (MIM: 608400), and Leber congenital amaurosis (MIM: 610142).[49–52] We propose that our proof-of-concept study provides evidence to translate this therapeutic approach to FECD given the accessibility of the diseased tissue and the relatively delayed onset of disease, which provides a window of opportunity to identify at risk individuals with *TCF4* repeat expansions and prevent disease progression in pre-symptomatic individuals.

Supplemental Data

Supplemental Data include six figures and nine tables and can be found with this article online at https://doi.org/10.1016/j.ajhg.2018.02.010.

Acknowledgments

We would like to thank all affected individuals for participating in this research. We would also like to acknowledge Dr. Charles A. Thornton for generously providing the MBNL1 (A2764) antibody used in this study and Miracles for Sight Eye bank for providing control corneal tissue. We thank Prof. Andrew Webster and Dr. Valentina Cipriani for granting access to AMD DNA samples and Prof. Veronica van Heyningen for providing a critical appraisal of the manuscript. This work was funded by Fight for Sight Early Career Investigator Award (A.E.D.), Academy of Medical Sciences (A.E.D.), The National Institute for Health Research Biomedical Research Centre at Moorfields Eye Hospital National Health Service Foundation Trust and UCL Institute of Ophthalmology, Rosetrees Trust (A.E.D. and A.J.H.), Wellcome Trust (M.E.C.), and ProQR Therapeutics. L.D., P.S., and P.L. were supported by PROGRES-Q26/LF1 and GACR 17-12355S. L.D. and P.L. were further supported by UNCE 204064 and P.S. by GA UK 250361/2017 and SVV 260367/2017. A patent has been filed by ProQR with UK and European patent offices (1517565) describing a method of treating FECD. P.A. is a listed inventor on this patent. P.K., K.D., and P.A. are all employees of ProQR Therapeutics. This work was supported in part by a grant from ProQR Therapeutics.

Received: January 3, 2018
Accepted: February 14, 2018
Published: March 8, 2018

Web Resources

CellProfiler (Broad Institute), http://cellprofiler.org/
GeneMarker Software (SoftGenetics), https://softgenetics.com/GeneMarker.php
GraphPad Prism 6 software, https://www.graphpad.com/scientific-software/prism/
OMIM, http://www.omim.org/
R 3.4.1 statistical software base packages, https://cran.r-project.org/

References

1. Zoega, G.M., Fujisawa, A., Sasaki, H., Kubota, A., Sasaki, K., Kitagawa, K., and Jonasson, F. (2006). Prevalence and risk factors for cornea guttata in the Reykjavik Eye Study. Ophthalmology *113*, 565–569.
2. Kitagawa, K., Kojima, M., Sasaki, H., Shui, Y.B., Chew, S.J., Cheng, H.M., Ono, M., Morikawa, Y., and Sasaki, K. (2002). Prevalence of primary cornea guttata and morphology of corneal endothelium in aging Japanese and Singaporean subjects. Ophthalmic Res. *34*, 135–138.
3. Lorenzetti, D.W., Uotila, M.H., Parikh, N., and Kaufman, H.E. (1967). Central cornea guttata. Incidence in the general population. Am. J. Ophthalmol. *64*, 1155–1158.
4. Vedana, G., Villarreal, G., Jr., and Jun, A.S. (2016). Fuchs endothelial corneal dystrophy: current perspectives. Clin. Ophthalmol. *10*, 321–330.
5. Eghrari, A.O., Riazuddin, S.A., and Gottsch, J.D. (2015). Fuchs corneal dystrophy. Prog. Mol. Biol. Transl. Sci. *134*, 79–97.
6. Goldberg, R.A., Raza, S., Walford, E., Feuer, W.J., and Goldberg, J.L. (2014). Fuchs endothelial corneal dystrophy: clinical characteristics of surgical and nonsurgical patients. Clin. Ophthalmol. *8*, 1761–1766.
7. Golchet, G., Carr, J., and Harris, M.G. (2000). Why don't we have enough cornea donors? A literature review and survey. Optometry *71*, 318–328.
8. Baratz, K.H., Tosakulwong, N., Ryu, E., Brown, W.L., Branham, K., Chen, W., Tran, K.D., Schmid-Kubista, K.E., Heckenlively, J.R., Swaroop, A., et al. (2010). E2-2 protein and Fuchs's corneal dystrophy. N. Engl. J. Med. *363*, 1016–1024.
9. Wieben, E.D., Aleff, R.A., Tosakulwong, N., Butz, M.L., Highsmith, W.E., Edwards, A.O., and Baratz, K.H. (2012). A common trinucleotide repeat expansion within the transcription factor 4 (TCF4, E2-2) gene predicts Fuchs corneal dystrophy. PLoS ONE *7*, e49083.
10. Scoles, D.R., Meera, P., Schneider, M.D., Paul, S., Dansithong, W., Figueroa, K.P., Hung, G., Rigo, F., Bennett, C.F., Otis, T.S., and Pulst, S.M. (2017). Antisense oligonucleotide therapy for spinocerebellar ataxia type 2. Nature *544*, 362–366.
11. Soliman, A.Z., Xing, C., Radwan, S.H., Gong, X., and Mootha, V.V. (2015). Correlation of severity of Fuchs endothelial corneal dystrophy with triplet repeat e'xpansion in TCF4. JAMA Ophthalmol. *133*, 1386–1391.
12. Vasanth, S., Eghrari, A.O., Gapsis, B.C., Wang, J., Haller, N.F., Stark, W.J., Katsanis, N., Riazuddin, S.A., and Gottsch, J.D. (2015). Expansion of CTG18.1 trinucleotide repeat in TCF4 is a potent driver of Fuchs' corneal dystrophy. Invest. Ophthalmol. Vis. Sci. *56*, 4531–4536.
13. Xing, C., Gong, X., Hussain, I., Khor, C.C., Tan, D.T., Aung, T., Mehta, J.S., Vithana, E.N., and Mootha, V.V. (2014). Transethnic replication of association of CTG18.1 repeat expansion of TCF4 gene with Fuchs' corneal dystrophy in Chinese implies common causal variant. Invest. Ophthalmol. Vis. Sci. *55*, 7073–7078.
14. Mootha, V.V., Gong, X., Ku, H.C., and Xing, C. (2014). Association and familial segregation of CTG18.1 trinucleotide repeat expansion of TCF4 gene in Fuchs' endothelial corneal dystrophy. Invest. Ophthalmol. Vis. Sci. *55*, 33–42.
15. Du, J., Aleff, R.A., Soragni, E., Kalari, K., Nie, J., Tang, X., Davila, J., Kocher, J.P., Patel, S.V., Gottesfeld, J.M., et al. (2015). RNA toxicity and missplicing in the common eye disease fuchs endothelial corneal dystrophy. J. Biol. Chem. *290*, 5979–5990.
16. Mahadevan, M., Tsilfidis, C., Sabourin, L., Shutler, G., Amemiya, C., Jansen, G., Neville, C., Narang, M., Barceló, J., O'Hoy, K., et al. (1992). Myotonic dystrophy mutation: an unstable CTG repeat in the 3′ untranslated region of the gene. Science *255*, 1253–1255.
17. Taneja, K.L., McCurrach, M., Schalling, M., Housman, D., and Singer, R.H. (1995). Foci of trinucleotide repeat transcripts in nuclei of myotonic dystrophy cells and tissues. J. Cell Biol. *128*, 995–1002.
18. Miller, J.W., Urbinati, C.R., Teng-Umnuay, P., Stenberg, M.G., Byrne, B.J., Thornton, C.A., and Swanson, M.S. (2000). Recruitment of human muscleblind proteins to (CUG)(n) expansions associated with myotonic dystrophy. EMBO J. *19*, 4439–4448.
19. Holt, I., Jacquemin, V., Fardaei, M., Sewry, C.A., Butler-Browne, G.S., Furling, D., Brook, J.D., and Morris, G.E. (2009). Muscleblind-like proteins: similarities and differences in normal and myotonic dystrophy muscle. Am. J. Pathol. *174*, 216–227.
20. Ciesiolka, A., Jazurek, M., Drazkowska, K., and Krzyzosiak, W.J. (2017). Structural characteristics of simple RNA repeats associated with disease and their deleterious protein interactions. Front. Cell. Neurosci. *11*, 97.
21. Charizanis, K., Lee, K.Y., Batra, R., Goodwin, M., Zhang, C., Yuan, Y., Shiue, L., Cline, M., Scotti, M.M., Xia, G., et al. (2012). Muscleblind-like 2-mediated alternative splicing in the developing brain and dysregulation in myotonic dystrophy. Neuron *75*, 437–450.
22. Du, H., Cline, M.S., Osborne, R.J., Tuttle, D.L., Clark, T.A., Donohue, J.P., Hall, M.P., Shiue, L., Swanson, M.S., Thornton, C.A., and Ares, M., Jr. (2010). Aberrant alternative splicing and extracellular matrix gene expression in mouse models of -myotonic dystrophy. Nat. Struct. Mol. Biol. *17*, 187–193.
23. Mulders, S.A., van den Broek, W.J., Wheeler, T.M., Croes, H.J., van Kuik-Romeijn, P., de Kimpe, S.J., Furling, D., Platenburg, G.J., Gourdon, G., Thornton, C.A., et al. (2009). Triplet-repeat oligonucleotide-mediated reversal of RNA toxicity in myotonic dystrophy. Proc. Natl. Acad. Sci. USA *106*, 13915–13920.
24. González-Barriga, A., Mulders, S.A., van de Giessen, J., Hooijer, J.D., Bijl, S., van Kessel, I.D., van Beers, J., van Deutekom, J.C., Fransen, J.A., Wieringa, B., and Wansink, D.G. (2013). Design and analysis of effects of triplet repeat oligonucleotides in cell models for myotonic dystrophy. Mol. Ther. Nucleic Acids *2*, e81.
25. Peh, G.S., Chng, Z., Ang, H.P., Cheng, T.Y., Adnan, K., Seah, X.Y., George, B.L., Toh, K.P., Tan, D.T., Yam, G.H., et al. (2015). Propagation of human corneal endothelial cells: a novel dual media approach. Cell Transplant. *24*, 287–304.

26. Carter, D.A., Nommiste, B., Coffey, P.J., and Carr, A.-J.F. (2016). Spontaneous generation of patient-specific retinal pigment epithelial cells using induced pluripotent stem cell technology. In Working with Stem Cells (Cham: Springer International Publishing), pp. 143–161.
27. Lin, X., Miller, J.W., Mankodi, A., Kanadia, R.N., Yuan, Y., Moxley, R.T., Swanson, M.S., and Thornton, C.A. (2006). Failure of MBNL1-dependent post-natal splicing transitions in myotonic dystrophy. Hum. Mol. Genet. *15*, 2087–2097.
28. Carpenter, A.E., Jones, T.R., Lamprecht, M.R., Clarke, C., Kang, I.H., Friman, O., Guertin, D.A., Chang, J.H., Lindquist, R.A., Moffat, J., et al. (2006). CellProfiler: image analysis software for identifying and quantifying cell phenotypes. Genome Biol. *7*, R100.
29. Afshari, N.A., Igo, R.P., Jr., Morris, N.J., Stambolian, D., Sharma, S., Pulagam, V.L., Dunn, S., Stamler, J.F., Truitt, B.J., Rimmler, J., et al. (2017). Genome-wide association study identifies three novel loci in Fuchs endothelial corneal dystrophy. Nat. Commun. *8*, 14898.
30. He, Z., Forest, F., Gain, P., Rageade, D., Bernard, A., Acquart, S., Peoc'h, M., Defoe, D.M., and Thuret, G. (2016). 3D map of the human corneal endothelial cell. Sci. Rep. *6*, 29047.
31. Bartakova, A., Alvarez-Delfin, K., Weisman, A.D., Salero, E., Raffa, G.A., Merkhofer, R.M., Jr., Kunzevitzky, N.J., and Goldberg, J.L. (2016). Novel identity and functional markers for human corneal endothelial cells. Invest. Ophthalmol. Vis. Sci. *57*, 2749–2762.
32. Ding, V., Chin, A., Peh, G., Mehta, J.S., and Choo, A. (2014). Generation of novel monoclonal antibodies for the enrichment and characterization of human corneal endothelial cells (hCENC) necessary for the treatment of corneal endothelial blindness. MAbs *6*, 1439–1452.
33. Peh, G.S., Toh, K.P., Ang, H.P., Seah, X.Y., George, B.L., and Mehta, J.S. (2013). Optimization of human corneal endothelial cell culture: density dependency of successful cultures in vitro. BMC Res. Notes *6*, 176.
34. Peh, G.S., Toh, K.P., Wu, F.Y., Tan, D.T., and Mehta, J.S. (2011). Cultivation of human corneal endothelial cells isolated from paired donor corneas. PLoS ONE *6*, e28310.
35. Mootha, V.V., Hussain, I., Cunnusamy, K., Graham, E., Gong, X., Neelam, S., Xing, C., Kittler, R., and Petroll, W.M. (2015). TCF4 triplet repeat expansion and nuclear RNA foci in Fuchs' endothelial corneal dystrophy. Invest. Ophthalmol. Vis. Sci. *56*, 2003–2011.
36. Konieczny, P., Stepniak-Konieczna, E., and Sobczak, K. (2017). MBNL expression in autoregulatory feedback loops. RNA Biol. *15*, 1–8.
37. Wieben, E.D., Aleff, R.A., Tang, X., Butz, M.L., Kalari, K.R., Highsmith, E.W., Jen, J., Vasmatzis, G., Patel, S.V., Maguire, L.J., et al. (2017). Trinucleotide repeat expansion in the transcription factor 4 (TCF4) gene leads to widespread mRNA splicing changes in Fuchs' endothelial corneal dystrophy. Invest. Ophthalmol. Vis. Sci. *58*, 343–352.
38. Gérard, X., Perrault, I., Munnich, A., Kaplan, J., and Rozet, J.M. (2015). Intravitreal injection of splice-switching oligonucleotides to manipulate splicing in retinal cells. Mol. Ther. Nucleic Acids *4*, e250.
39. Schoch, K.M., and Miller, T.M. (2017). Antisense oligonucleotides: translation from mouse models to human neurodegenerative diseases. Neuron *94*, 1056–1070.
40. Jiang, J., Zhu, Q., Gendron, T.F., Saberi, S., McAlonis-Downes, M., Seelman, A., Stauffer, J.E., Jafar-Nejad, P., Drenner, K., Schulte, D., et al. (2016). Gain of toxicity from ALS/FTD-linked repeat expansions in C9ORF72 is alleviated by antisense oligonucleotides targeting GGGGCC-containing RNAs. Neuron *90*, 535–550.
41. Wheeler, T.M., Leger, A.J., Pandey, S.K., MacLeod, A.R., Nakamori, M., Cheng, S.H., Wentworth, B.M., Bennett, C.F., and Thornton, C.A. (2012). Targeting nuclear RNA for in vivo correction of myotonic dystrophy. Nature *488*, 111–115.
42. Kordasiewicz, H.B., Stanek, L.M., Wancewicz, E.V., Mazur, C., McAlonis, M.M., Pytel, K.A., Artates, J.W., Weiss, A., Cheng, S.H., Shihabuddin, L.S., et al. (2012). Sustained therapeutic reversal of Huntington's disease by transient repression of huntingtin synthesis. Neuron *74*, 1031–1044.
43. Gipson, I.K. (2013). Age-related changes and diseases of the ocular surface and cornea. Invest. Ophthalmol. Vis. Sci. *54*, ORSF48-53.
44. Amiel, J., Rio, M., de Pontual, L., Redon, R., Malan, V., Boddaert, N., Plouin, P., Carter, N.P., Lyonnet, S., Munnich, A., and Colleaux, L. (2007). Mutations in TCF4, encoding a class I basic helix-loop-helix transcription factor, are responsible for Pitt-Hopkins syndrome, a severe epileptic encephalopathy associated with autonomic dysfunction. Am. J. Hum. Genet. *80*, 988–993.
45. Zweier, C., Peippo, M.M., Hoyer, J., Sousa, S., Bottani, A., Clayton-Smith, J., Reardon, W., Saraiva, J., Cabral, A., Gohring, I., et al. (2007). Haploinsufficiency of TCF4 causes syndromal mental retardation with intermittent hyperventilation (Pitt-Hopkins syndrome). Am. J. Hum. Genet. *80*, 994–1001.
46. Mootha, V.V., Hansen, B., Rong, Z., Mammen, P.P., Zhou, Z., Xing, C., and Gong, X. (2017). Fuchs' endothelial corneal dystrophy and RNA foci in patients with myotonic dystrophy. Invest. Ophthalmol. Vis. Sci. *58*, 4579–4585.
47. Jain, A., and Vale, R.D. (2017). RNA phase transitions in repeat expansion disorders. Nature *546*, 243–247.
48. Henry, S.P., Miner, R.C., Drew, W.L., Fitchett, J., York-Defalco, C., Rapp, L.M., and Levin, A.A. (2001). Antiviral activity and ocular kinetics of antisense oligonucleotides designed to inhibit CMV replication. Invest. Ophthalmol. Vis. Sci. *42*, 2646–2651.
49. Murray, S.F., Jazayeri, A., Matthes, M.T., Yasumura, D., Yang, H., Peralta, R., Watt, A., Freier, S., Hung, G., Adamson, P.S., et al. (2015). Allele-specific inhibition of rhodopsin with an antisense oligonucleotide slows photoreceptor cell degeneration. Invest. Ophthalmol. Vis. Sci. *56*, 6362–6375.
50. Lentz, J.J., Jodelka, F.M., Hinrich, A.J., McCaffrey, K.E., Farris, H.E., Spalitta, M.J., Bazan, N.G., Duelli, D.M., Rigo, F., and Hastings, M.L. (2013). Rescue of hearing and vestibular function by antisense oligonucleotides in a mouse model of human deafness. Nat. Med. *19*, 345–350.
51. Garanto, A., Chung, D.C., Duijkers, L., Corral-Serrano, J.C., Messchaert, M., Xiao, R., Bennett, J., Vandenberghe, L.H., and Collin, R.W. (2016). In vitro and in vivo rescue of aberrant splicing in CEP290-associated LCA by antisense oligonucleotide delivery. Hum. Mol. Genet. *25*, 2552–2563.
52. Parfitt, D.A., Lane, A., Ramsden, C.M., Carr, A.J., Munro, P.M., Jovanovic, K., Schwarz, N., Kanuga, N., Muthiah, M.N., Hull, S., et al. (2016). Identification and correction of mechanisms underlying inherited blindness in human iPSC-derived optic cups. Cell Stem Cell *18*, 769–781.

The American Journal of Human Genetics, Volume *102*

Supplemental Data

Antisense Therapy for a Common Corneal Dystrophy Ameliorates *TCF4* Repeat Expansion-Mediated Toxicity

Christina Zarouchlioti, Beatriz Sanchez-Pintado, Nathaniel J. Hafford Tear, Pontus Klein, Petra Liskova, Kalyan Dulla, Ma'ayan Semo, Anthony A. Vugler, Kirithika Muthusamy, Lubica Dudakova, Hannah J. Levis, Pavlina Skalicka, Pirro Hysi, Michael E. Cheetham, Stephen J. Tuft, Peter Adamson, Alison J. Hardcastle, and Alice E. Davidson

Supplemental Data

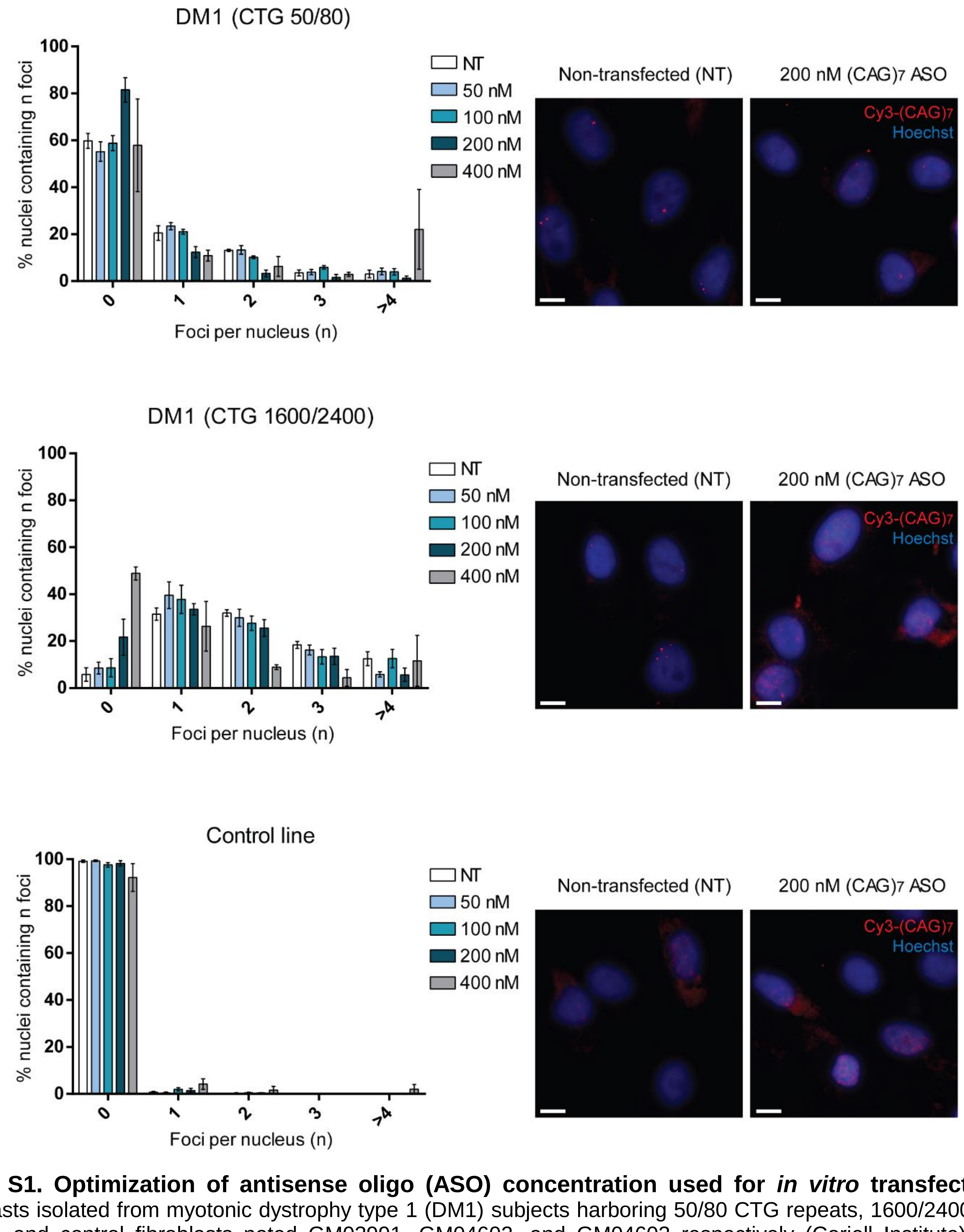

Figure S1. Optimization of antisense oligo (ASO) concentration used for *in vitro* transfections. Fibroblasts isolated from myotonic dystrophy type 1 (DM1) subjects harboring 50/80 CTG repeats, 1600/2400 CTG repeats, and control fibroblasts noted GM03991, GM04602, and GM04603 respectively (Coriell Institute) were transfected with 2'Ome-PS-$(CAG)_7$ ASO at indicated concentrations using Turbofect (ThermoFisher Scientific). Twenty-four hours post transfection fluorescence *in situ* hybridization (FISH) was performed and RNA foci were quantified using CellProfiler (Broad Institute). A concentration of 200 nM 2'Ome-PS-$(CAG)_7$ ASO was selected to be optimal, on the basis for these data, for future ASO transfection experiments in corneal endothelial cells (CECs). Scale bars, 10 µm.

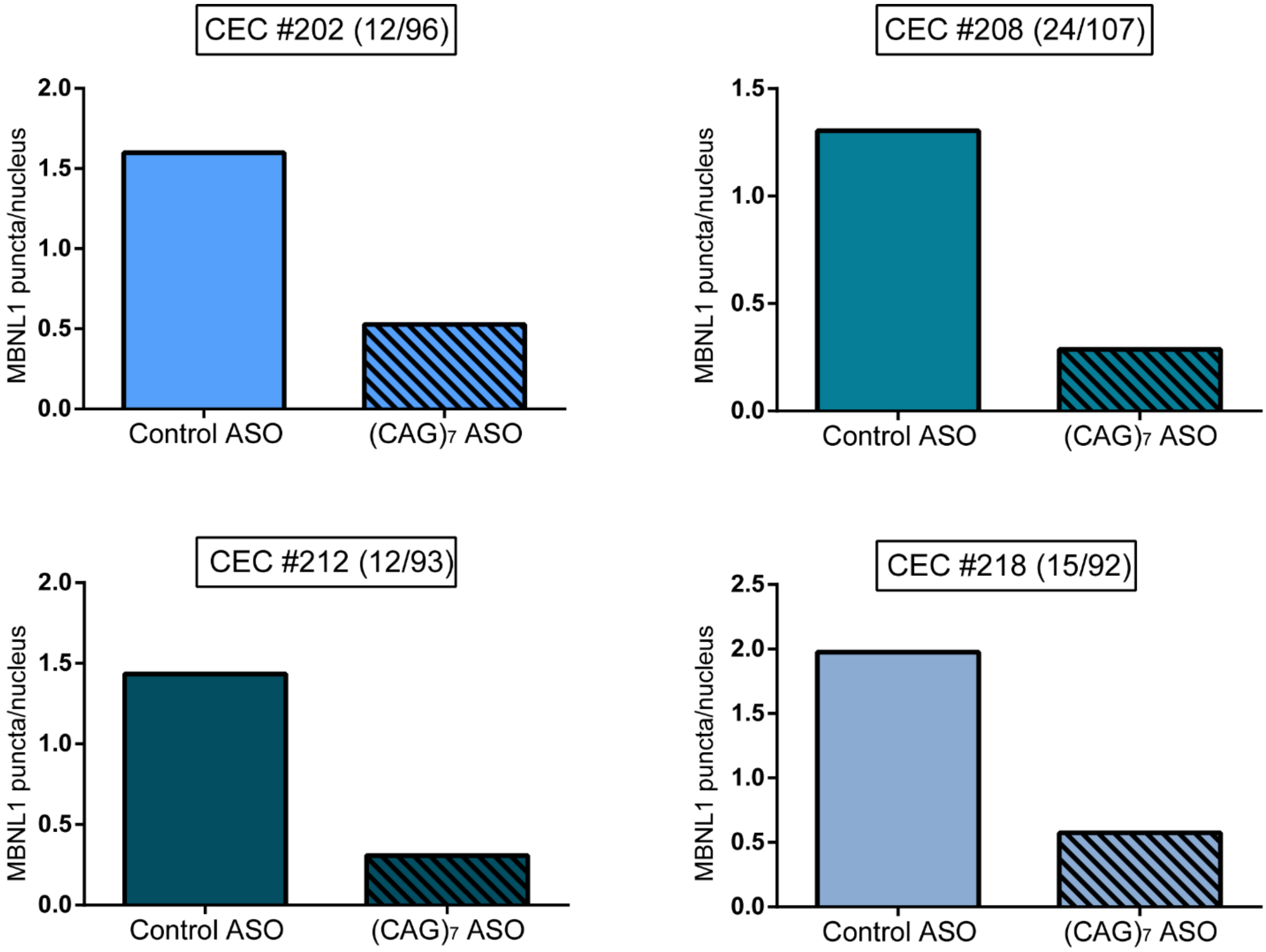

Figure S2. Quantification of nuclear MBNL1-positive puncta post antisense oligo (ASO) treatments. The result of 4 independent Fuchs endothelial corneal dystrophy (FECD) case-derived CEC lines, where the numbers of MBNL1 puncta were quantified after treatment with the control and $(CAG)_7$ ASOs. CTG18.1 genotypes are shown in parentheses for each line investigated.

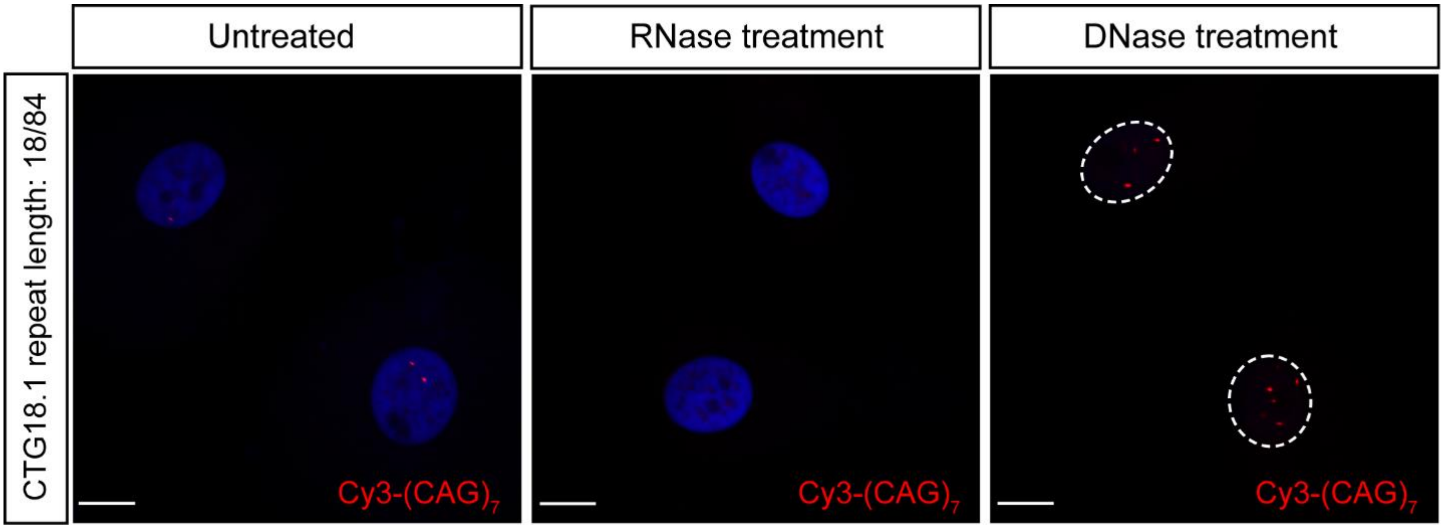

Figure S3. Fluorescence *in situ* hybridization (FISH) protocol detects RNA-specific CUG foci. Sense RNA foci detection with Cy3-$(CAG)_7$ probe in untreated, RNase treated and DNase treated FECD case-derived CECs with an expanded *TCF4* CTG18.1 allele (18/84). Scale bars, 10 µm.

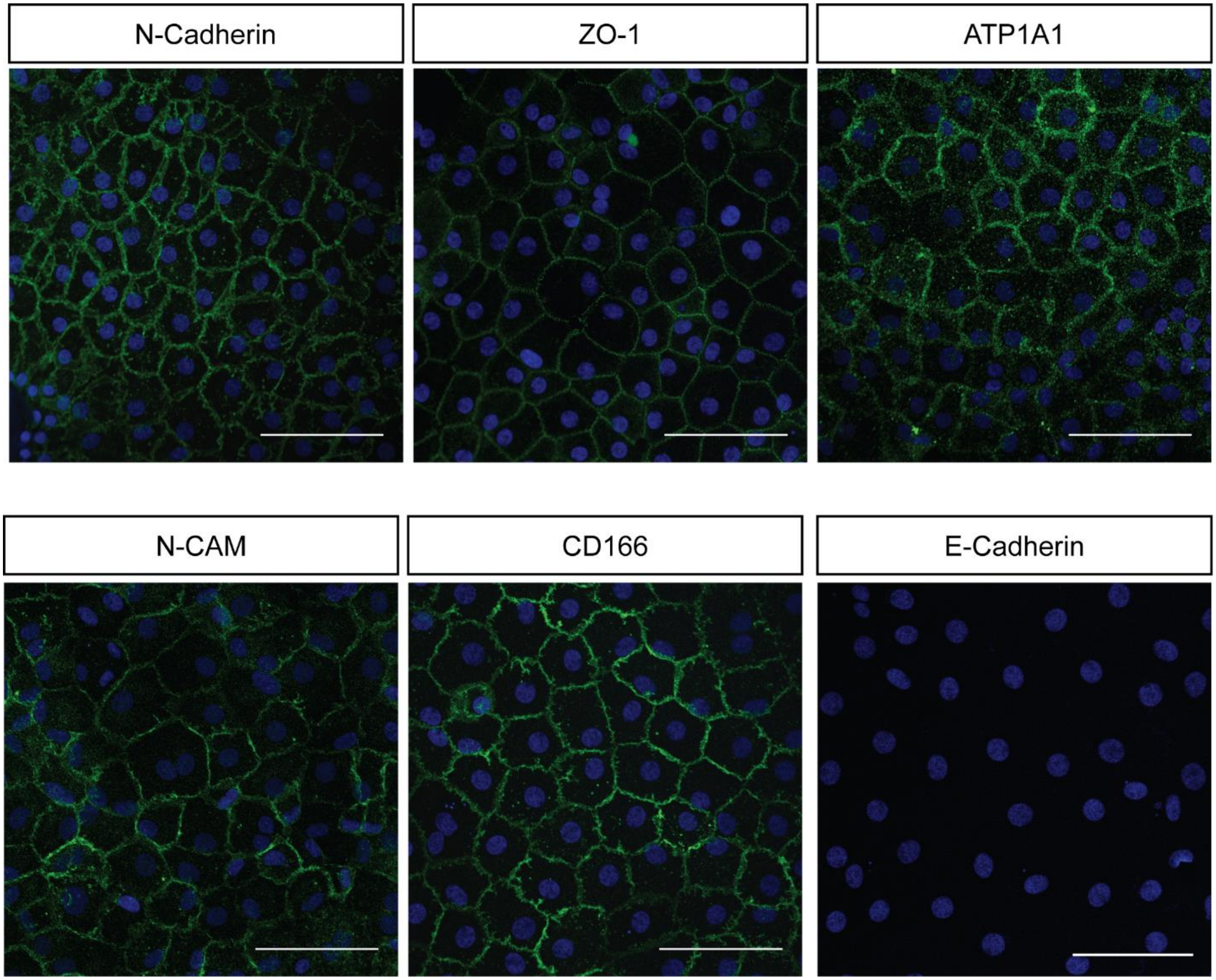

Figure S4. Primary corneal endothelial cell (CEC) cultures display distinctive corneal endothelial polygonal morphology and display markers indicative of endothelial cell status. Endothelial markers N-Cadherin, ZO-1, ATP1A1, N-CAM and CD166 were observed and the epithelial marker E-Cadherin was absent in CEC lines derived from healthy tissue. Scale bars, 100 µm.

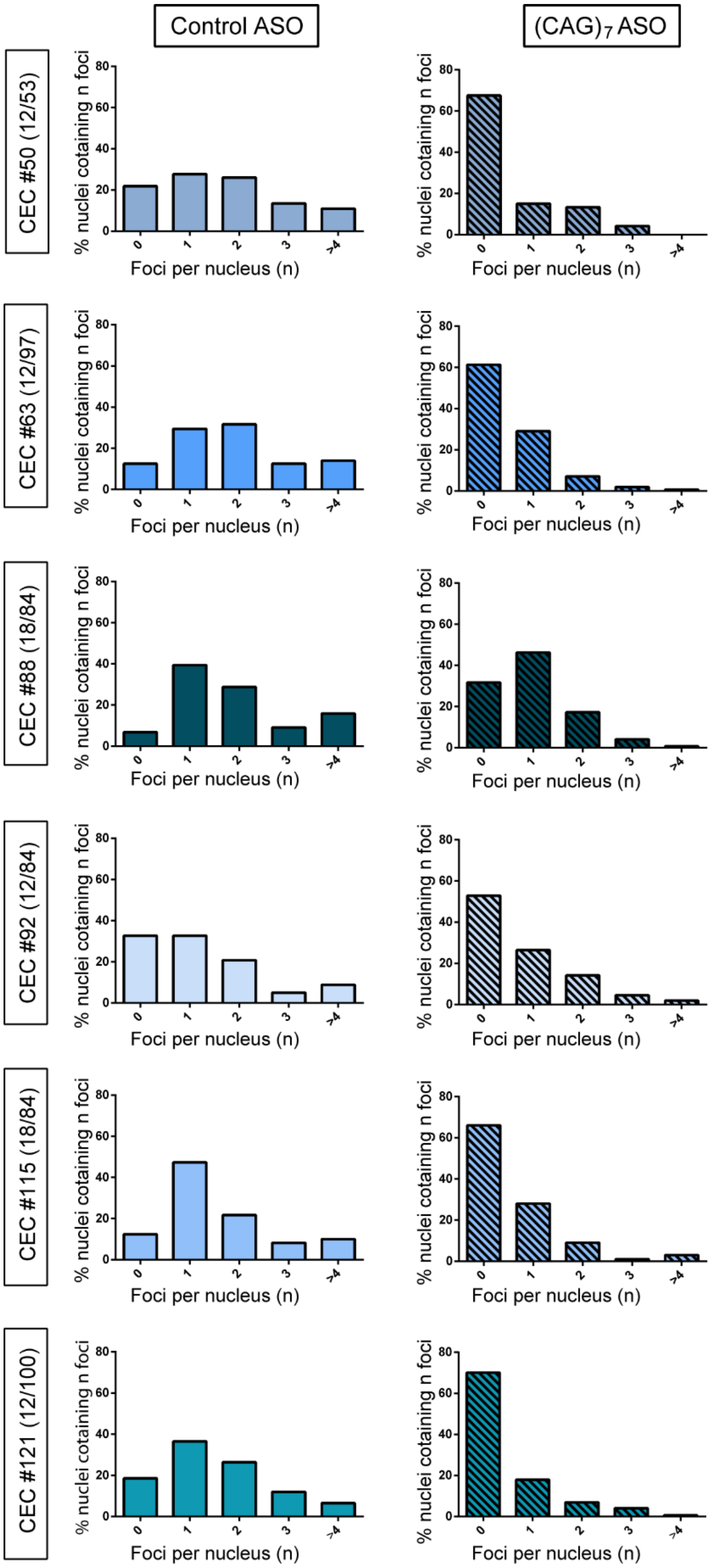

Figure S5. Antisense oligo (ASO)-mediated treatment of 6 independent FECD case-derived corneal endothelial cell (CEC) lines. The effect of the $(CAG)_7$ ASO treatment on foci incidence in each of the 6 independent FECD case-derived cell lines investigated is shown. CTG18.1 genotypes are shown in parentheses for each line investigated.

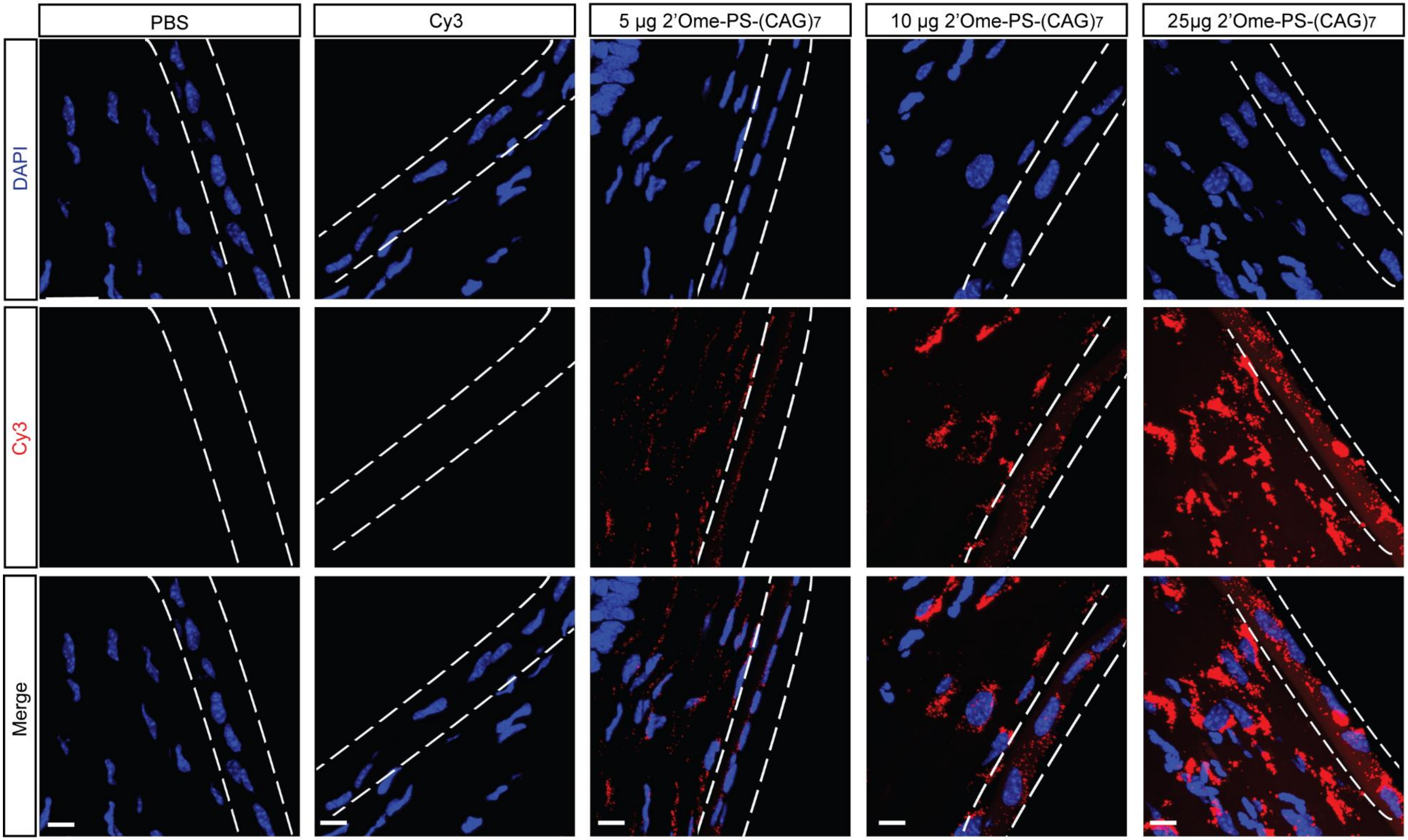

Figure S6. Visualization of Cy3-labelled 2’Ome-PS-(CAG)$_7$ antisense oligonucleotide (ASO) in corneal tissue following intraocular administration. C57Bl6 mice were intravitreally injected with 0.025, 0.01 or 0.005 mg of Cy-3 labelled 2’Ome-PS-(CAG)$_7$ ASO, free Cy3 dye alone (identical molar amount to the ASO), or PBS. Animals were sacrificed 48h post injection. Eyes were enucleated and immersion fixed in paraformaldehyde. Slides were prepared and immediately viewed on a confocal fluorescent microscope after counterstaining for cell nuclei detection with DAPI (blue). The location of the corneal endothelium is denoted by dashed lines. Scale bars, 20 µm.

**Table S1. Summary of antibodies used in this study.**			
**Target protein**	**Source**	**Dilution**	**Reference**
ZO-1	Mouse	1:500	Invitrogen
ATP1A1	Mouse	1:50	Developmental studies Hybridoma Bank (a6F)
N-Cadherin	Rabbit	1:100	Abcam (ab12221)
N-CAM	Rabbit	1:100	Cell signaling 3606s
CD166	Mouse	1:200	Pharmingen (BD) 559260
E-Cadherin	Rat	1:500	Abcam (ab11512)
MBNL1	Rabbit	1:2000	Lin et al, 2006 [27]
MBNL2	Mouse	1:100	Santa Cruz (sc-365104)

**Table S2. Primers used to detect alternative splice events by reverse transcriptase (RT)-PCR.**			
**Primer**	**Sequence 5'-3'**	**Amplicon sizes (bp)**	**Optimized annealing temperature (^{0}C)**
*MBNL1*_F	TGACACCAATGACAACACAGTC	383/329	65
*MBNL1*_ R	CTGGTGGGAGAAATGCTGTA		
*MBNL2*_ F	ACAGCACCATGATCGACACAAG	276/222	64
MBNL2 _R	AAGGATGAAGAGCACCAGGG		
*NUMA1*_F	TACGTGCTGATGCTGAGACC	305/263	64
*NUMA1*_R	CTTGCTGGCTTGGTCAGAGT		

Table S3: Fibroblast, and case matched corneal endothelial cell (CEC), lines investigated by fluorescence *in situ* hybridization (FISH). NA, not available. NI, not investigated. CTG18.1 repeat lengths were confirmed to be concordant between blood and fibroblast-derived gDNA samples.

Fibroblast line	Case matched CEC line	CTG18.1 genotype	CUG RNA foci detected in fibroblasts?	CUG RNA foci detected in CECs?
#F1	NA	31/69	No	NI
#F2	NA	12/53	No	NI
#F3	NA	84/108	No	NI
#F4	#43	23/68	No	Yes
#F5	#47	23/70	No	Yes
#F6	#31	12/77	No	Yes

Table S4: CTG18.1 genotype of FECD case-derived corneal endothelial cell (CEC) lines investigated by fluorescence *in situ* hybridization (FISH). Cell lines are ordered based on CTG18.1 genotype status in ascending order. Genotypes were generated using case matched whole blood-derived genomic DNA samples.

CEC line	CTG18.1 genotype	CUG RNA foci detected?
#94	12/12	No
#139	12/12	No
#42	12/15	No
#86	12/15	No
#32	12/16	No
#46	26/27	No
#190	15/16	No
#211	12/23	No
#61	18/31	No
#181	25/31	Yes
#50	12/53	Yes
#136	12/58	Yes
#8	16/62	Yes
#38	16/62	Yes
#152	15/68	Yes
#43	23/68	Yes
#33	16/70	Yes
#47	23/70	Yes
#120	23/70	Yes
#70	12/74	Yes
#49	12/76	Yes
#192	25/76	Yes
#31	12/77	Yes
#193	25/79	Yes
#25	24/80	Yes
#29	16/81	Yes
#37	12/82	Yes
#92	12/84	Yes
#88	18/84	Yes
#115	18/84	Yes
#34	12/87	Yes
#137	16/88	Yes
#63	12/97	Yes
#67	12/98	Yes
#121	12/100	Yes
#44	12/126	Yes

Table S5: FECD case-derived corneal endothelial cell (CEC) lines used for dual fluorescence *in situ* hybridization (FISH) and immunocytochemistry (ICC) experiments. NI, not investigated, E, expanded, NE, non-expanded.

Case derived CEC line	CTG18.1 genotype	CTG18.1 Genotype Classification	CUG RNA foci detected?	MBNL1 sequestered?	MBNL2 sequestered?
#121	12/100	E	Yes	Yes	NI
#190	15/16	NE	No	No	NI
#192	25/76	E	Yes	Yes	NI
#193	25/79	E	Yes	Yes	NI
#204	12/99	E	Yes	NI	Yes
#206	25/76	E	Yes	NI	Yes
#211	12/23	NE	No	NI	No
#226	12/18	NE	No	No	NI

Table S6: FECD case-derived corneal endothelial cell (CEC) lines used for transcriptomic analysis. E, expanded, NE, non-expanded.

Case matched CEC line	CTG18.1 genotype	CTG18.1 Genotype Classification
#138	16/84	E
#140	12/106	E
#149	16/93	E
#154	12/81	E
#125	13/18	NE
#141	18/24	NE
#145	22/28	NE

Table S7: FECD case-derived corneal endothelial cell (CEC) lines used for antisense oligonucleotide (ASO) treatment experiments. E, expanded.

Case matched CEC line	CTG18.1 genotype	CTG18.1 Genotype Classification
#88	18/84	E
#92	12/84	E
#115	18/84	E
#121	12/100	E
#50	12/53	E
#63	12/97	E

Table S8: FECD case-derived corneal endothelial cell (CEC) lines used to assess MBNL1 localization, following antisense oligonucleotide (ASO) treatment. E, expanded.

Case matched CEC line	CTG18.1 genotype	CTG18.1 Genotype Classification
#202	12/96	E
#208	24/107	E
#212	12/93	E
#218	15/92	E

Table S9: FECD case-derived corneal endothelial cell (CEC) lines used for transcriptomic analysis, following antisense oligonucleotide (ASO) treatment. E, expanded.

Case matched CEC line	CTG18.1 genotype	CTG18.1 Genotype Classification
#162	71/113	E
#173	16/72	E
#179	15/61	E
#210	14/80	E
#215	12/73	E
#216	18/91	E
#219	18/88	E
#220	15/103	E
#223	12/63	E
#224	12/89	E
